# Pigmented fungi derived from hot and cold deserts ecosystems: taxonomy, genomic and biotechnological approach

**DOI:** 10.1007/s11274-026-05160-0

**Published:** 2026-07-31

**Authors:** Mayanne Karla da Silva, Rubens Correia da Silva, Beatriz Medeiros de Souza Melo, Thiago José Carvalho Cavalcante, Leonardo da Silva Santos, João Gabriel Pereira e Silva, Vannêssa Rodrigues Teles Maia, Monelly da Silva Bernardo, Kelvin da Silva Sousa, Averlane Vieira da Silva, Adeildo Júnior de Oliveira, Aline Cavalcanti de Queiroz, Luiz Henrique Rosa, Alysson Wagner Fernandes Duarte

**Affiliations:** 1 Laboratório de Microbiologia, Imunologia e Parasitologia, Universidade Federal de Alagoas, Campus Arapiraca, Arapiraca, Alagoas Brazil; 2 Núcleo de Ciências Exatas, Universidade Federal de Alagoas, Arapiraca, Alagoas Brazil; 3 Laboratório de Farmacologia e Imunidade, Universidade Federal de Alagoas, Campus A.C Simões, Maceió, Alagoas Brazil; 4 Instituto de Ciências Biológicas, Universidade Federal de Minas Gerais, Belo Horizonte, Minas Gerais Brazil

**Keywords:** Extreme environments, Extremophiles, Culture collection, Colorants, Potential, Unlocking bioprospecting

## Abstract

Safer and more efficient products and processes are current global demands that drive the field of bioprospective research. Microbial sources are the main natural alternative solutions that have contributed to this biotechnological line, with environmental fungi representing important promising forces. Particularly, those inhabiting the most unexplored extreme geographical areas of the planet, marked by severe aridity, with hot and/or cold thermal profiles, e.g., the Atacama Desert, Saudi Arabia, Taklamakan Desert, semi-arid regions (Caatinga and Mexico), and polar and alpine ecosystems. In these habitats, many abiotic stressors are present, and fungi thrive through a combination of adaptations, including the production of pigmented secondary metabolites with diverse active biological functions that have high potential for innovative industrial applications, which is being explored in greater depth at the present time. In this sense, this article has aimed to review pigmented fungi from extreme arid environments from a taxonomic, genomic and biotechnological perspective. An attempt has been made to compile information on pigmented fungi in pure culture, their chromatic aspects, chemical classes, genomic data and application across diverse domains. There were more ascomycete and basidiomycete representatives producing a wide spectrum of endo- and extracellular colors categorized as melanins, carotenoids, and polyketides. Furthermore, they prove useful across multiple biotechnological sectors, ranging from agriculture, food, cosmetics, and medicine to astrobiology. Those with investigated genomes showed genes linked to the biosynthesis of these pigments and to environmental adaptation. Thus, with this initiative, we accessed the bioprospective importance of extremophilic colored fungi from arid habitats.

**Graphical Abstract:**

**Figure:**
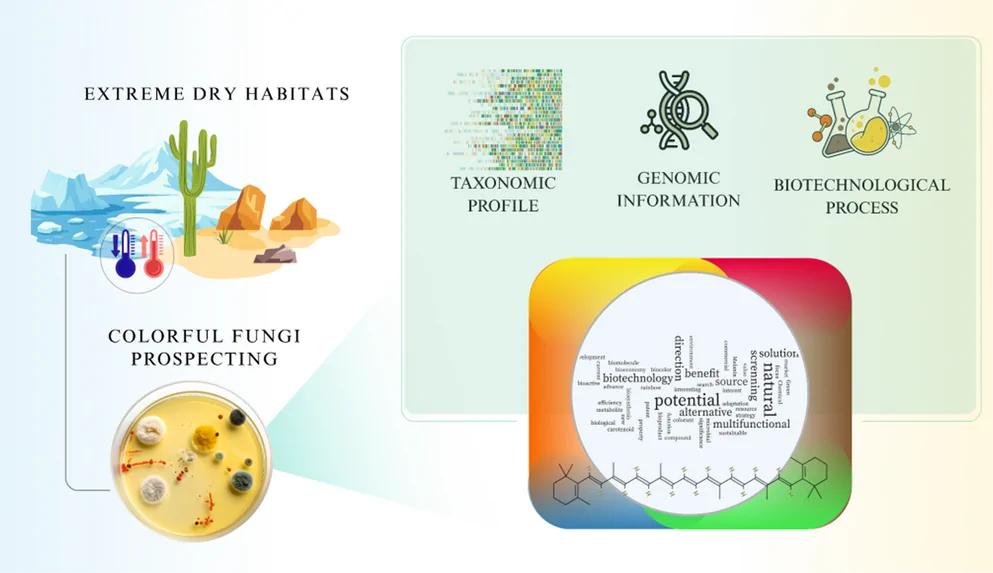

**Supplementary Information:**

The online version contains supplementary material available at https://doi.org/10.1007/s11274-026-05160-0.

## Introduction

Pigments are molecules that capture light, absorbing and reflecting it at different wavelengths, generally in the range of 200 to 800 nm, producing a specific color pattern as a result. Depending on their origin, they are commonly classified as natural or synthetic based on their origin, and as organic or inorganic based on their chemical composition (Barreto et al. 2023). Natural pigments are treated as the colored secondary metabolites produced by organisms from different domains of biological life, including plants, animals and microorganisms (Pasdaran et al. 2023; Tang et al. 2024). Despite these distinct origins, microbial pigments are more advantageous in terms of broad ecosystem adaptability, yield, production and potential uses, even when compared to their synthetic counterparts, due to the potential of their biological characteristics to confer, above all, safety and biodegradability (Chatragadda and Dufossé 2021; Barreto et al. 2023).

The biosynthesis of these types of molecules by microorganisms derived from extreme desert areas is one of the main adaptation strategies observed, playing a role in the systemic cellular response to environmentally imposed stressors involving intense thermal, energy, nutritional and/or light demands (Duarte et al. 2018a, b; Rao et al. 2021). Among the many harsh physical and chemical conditions, combined with temperature indices, the important extreme desert areas correspond to geographical zones that receive low levels of average precipitation throughout the year, generally below 600 mm, therefore marked by intense environmental aridity, a uniform parameter for both hot and cold deserts on the planet (Azua-Bustos et al. 2012; Makhalanyane et al. 2015; Arros et al. 2024). As natural niches concentrated on different continents of the planet, as arid regions have a high percentage of distribution in the total global area (more than 40%) and based on the aridity index (AI) estimated by the ratio between precipitation and annual evapotranspiration, there is a categorical zoning of these regions into hyper-arid, arid, semi-arid or dry subhumid, as well as cryospheric and/or polar habitats (Rodríguez-Núñez et al. 2020; Rodríguez-González et al. 2024). Interestingly, microorganisms are the life forms that thrive most in these extremely challenging dry habitats, directly impacting environmental balance through their unique ecological services (Makhalanyane et al. 2015). Furthermore, because they are demanding environments, they exert strong restrictive pressure capable of selecting the most specialized and tolerant microbial strain (Sterflinger et al. 2012; Han et al. 2024).

Globally dispersed and highly resilient, fungi are among the most diverse and successful eukaryotes residing in extreme ecosystems (Coleine et al. 2022). Surveys of prevalent lineages in those areas marked by aridity are increasing and favorably expanded by the robustness of geographic access technologies, cultivation methods, omics tools and the trend toward exploring unknown microbial dark matter in these large underexplored environments (Han et al. 2024). The high capacity for natural tolerance of fungi from these extreme areas can be reflected in their genomes, metabolic activities and morphological versatility (Rosa et al. 2019; Coleine et al. 2022; Vimercati et al. 2025), including the role of their secondary metabolites, focusing on the biosynthetic pathways of their pigments, along with other adaptive strategies that are not yet so clear at the molecular and genomic level (Gomez-Gutierrrez et al. 2024; Palma et al. 2024).

**Fig. 1:**
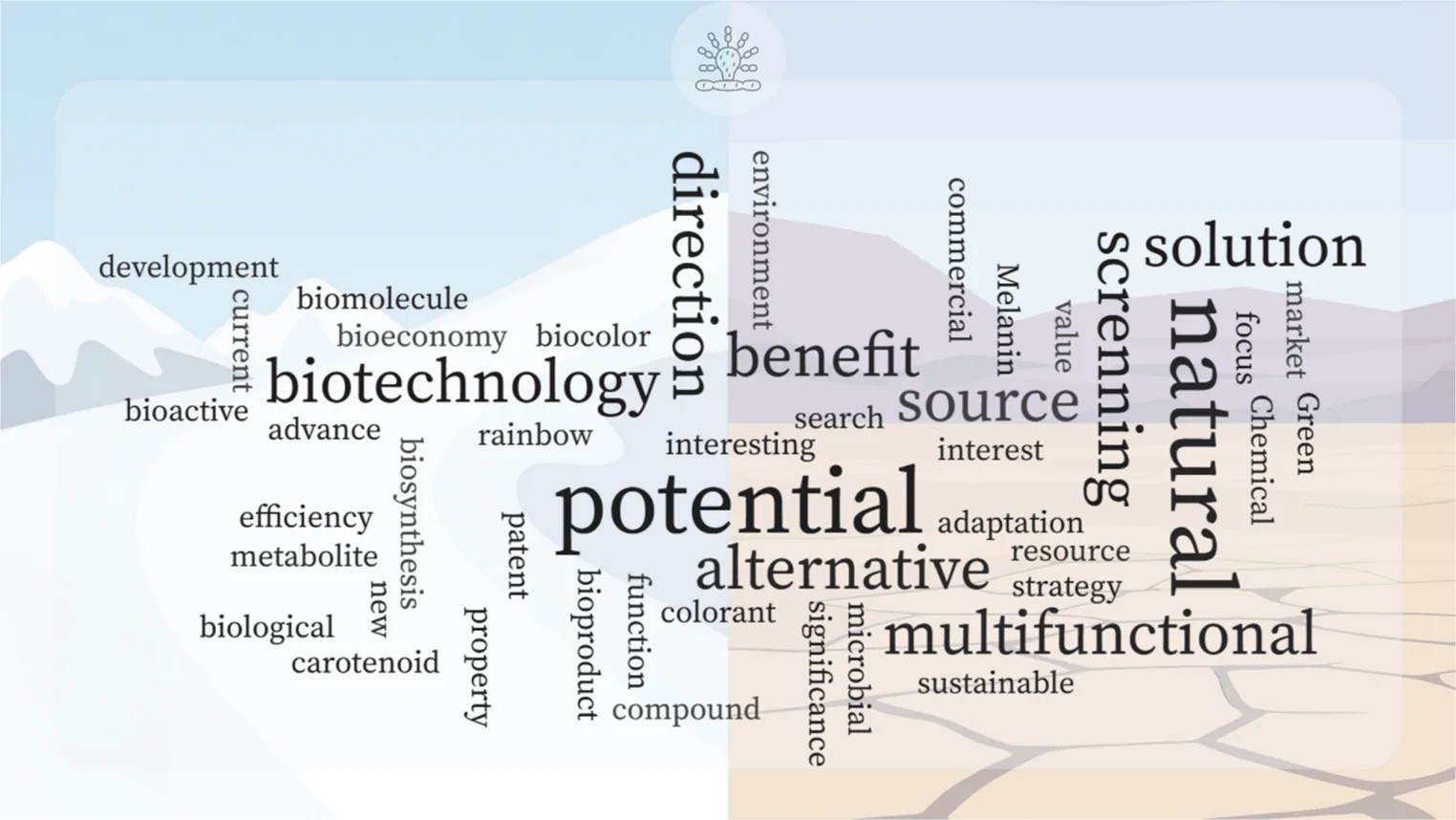
Terms related to fungal biopigments in the bioprospecting process with extreme desert ecosystems

The synthesis, accumulation, secretion and functions of pigmented molecules in fungi have been the focus of much research (Hasanien et al. 2022; Aliyu et al. 2025), including the growing interest among extremophiles from desert habitats (Jiang et al. 2020; Cavalcante et al. 2024; Alyami et al. 2025). The aim is to understand not only the mechanisms involved in these processes, but also to establish the systematic, chemotaxonomic, ecological, and biotechnological value of the producing fungal groups (Chatragadda and Dufossé 2021; Cavalcante et al. 2024). When it comes to the search for pigments and other molecules derived from environmental fungi, particularly those from drylands, recurring terms and expressions in the literature, when grouping them together, include positive screening, significant potential, sources of biological components, bioproducts and/or alternative, natural and promising metabolites, represent a hidden rainbow, with current and future direction, a new research focus, linked to the sustainable impact of their benefits, multifunctions and bioactivities, therefore interesting in the bioeconomy, for commercial/market use and in the ramifications of biotechnology (i.e.: blue, red, green and brown) **(**Fig. 1**)**.

With this contextualization and relevance of the topic discussed, the purpose of this narrative review was to summarize studies of adapted pigment-producing fungi reported from desert regions in arid and cold climate zones, the main pigmented strains cultivated, their color spectrum, chemical classes, biological properties, and potential for biotechnological use in the agrochemical, food, textile, medicinal/pharmaceutical, cosmetic, and environmental sectors, in addition to analyses of their genomic contents and the environmental bioprospecting steps related to the isolation and production processes of their natural pigments. A narrative review was carried out based on the most recent studies possible, within the last 10 to 15 years, found mainly in the Scopus, Science Direct, Google Scholar and PubMed databases, using different combinations of descriptors based on terms related to the proposed objectives. Based on an exploratory survey, the listed terms were gathered from review and original studies on pigmented fungi, subsequently used in the Mentimeter tool (https://www.mentimeter.com), and the final image was generated in Canva (https://www.canva.com). Figure 1.

### Taxonomic records of pigment-producing fungi from extreme environments: main strains, colors, chemical types and bioactivities

**Fig. 2:**
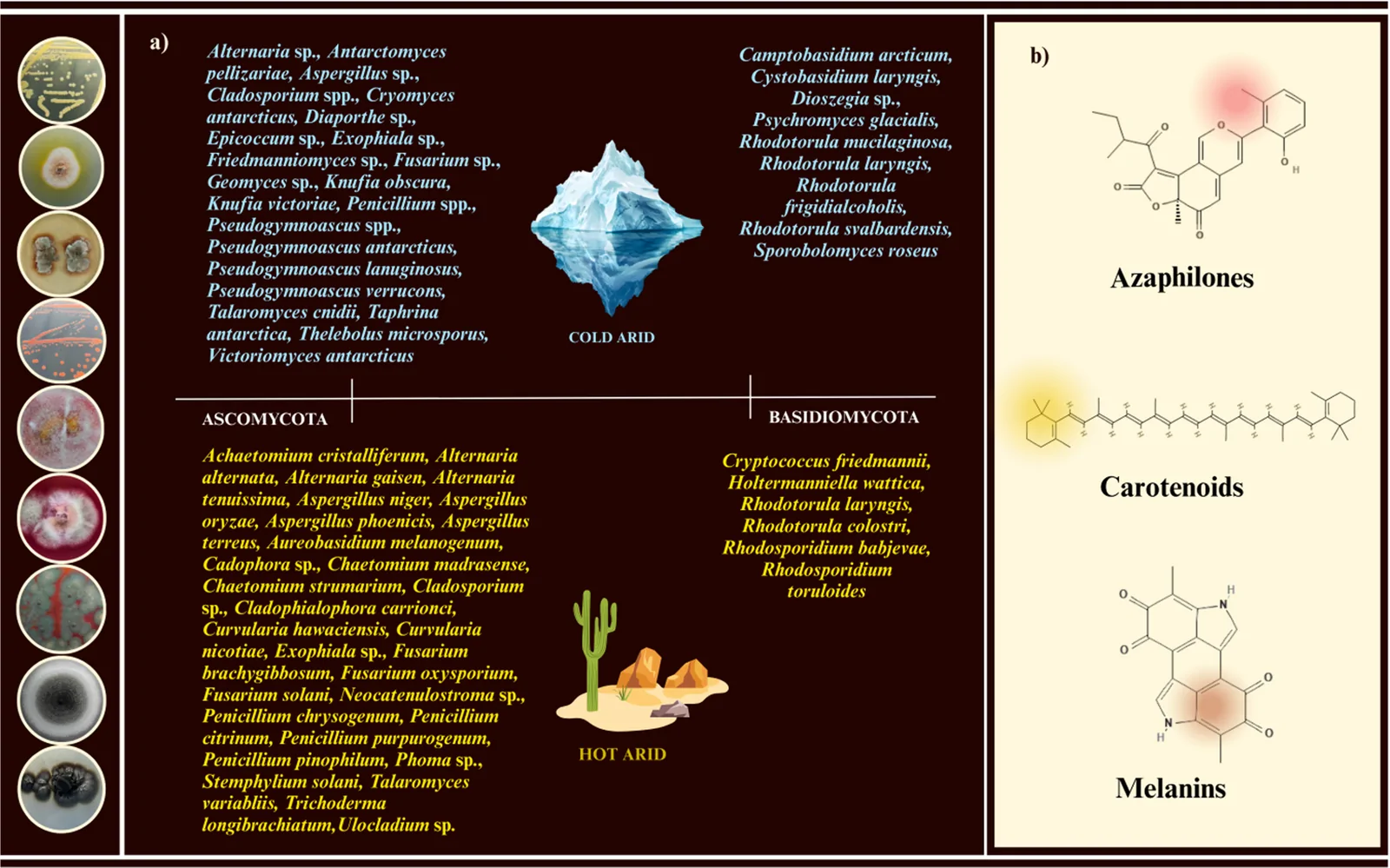
(**a**) Pigmented fungal taxa reported from cold and hot desert regions affiliated with the phyla Ascomycota and Basidiomycota; (**b**) Chemical categories and color spectrum of their biopigments

The objective of this topic was to gather taxa of colored fungi isolated from cryospheric and arid desert ecosystems, their original environmental substrates, including records of previously described genera and species, their predominant colors in colonies, the predominant pigmentation and the type of pigment, when studied. In addition to pigmentation, relevant bioactivities of the colored fungi were also collected, aiming to contribute to the understanding of the biology and overall potential of the colored strains, looking at the biotechnological possibilities of the reported taxa beyond the function of the colors of their colonies and their pigments (**Supplementary Table** 1). In the literature review, a prevalence of ascomycetic filamentous fungi was observed mainly representatives of the genera *Achaetomium*, *Alternaria*, *Antarctomyces*, *Aureobasidium*, *Aspergillus*, *Cadophora*, *Chaetomium*, *Cladosporium*, *Cryomyces*, *Curvularia*, *Diaporthe*, *Epicoccum*, *Exophiala*, *Friedmanniomyces*, *Fusarium*, *Geomyces*, *Knufia*, *Penicillium*, *Phoma*, *Pseudogymoascus*, *Talaromyces*, *Taphira*, *Thelebolus* and *Tricoderma*, along with some groups of pigmented basidiomycete yeasts belonging to the genera *Camptobasidium*, *Cryptococcus*, *Cystobasidium*, *Dioszegia*, *Holtermanniella*, *Psychromyces*, *Rhodosporidium*, *Rhodotorula* and *Sporobolomyces*. Categorically, the production of melanin, carotenoid, and polyketide pigments (of the azaphyllone type) was observed among the fungi in the reviewed studies (Fig. 2).

### Hot arid ecosystems

Fungal pigments play a fundamental role in the survival and ecological success of fungi inhabiting arid environments, which are characterized by intense solar radiation, high incidence of ultraviolet (UV) radiation, large daily temperature ranges, and low water availability (Santiago et al. 2018). In this ecological context, compounds such as melanins, carotenoids, and other pigmented secondary metabolites act as important cellular protection mechanisms (Elkhateeb and Daba 2023; Toma et al. 2023). The production of these pigments is not only related to coloration but also represents an adaptive strategy that increases tolerance to severe environmental stress (Cordero and Casadevall 2017). Among the most studied pigments, melanin stands out, considered one of the main protective factors in fungi exposed to extreme conditions. This pigment is deposited in the cell wall of hyphae and spores, where it acts as a physical and chemical barrier. Melanin has the ability to absorb and dissipate UV radiation, and by reducing damage to DNA and other sensitive cellular structures, decreasing radiation-induced mutations, in arid environments, where vegetation cover is scarce and sun exposure is intense, this property becomes particularly relevant for maintaining cell viability (Onoda et al. 2024; Chhoker et al. 2025; León et al. 2025).

In addition to radiation protection, fungal pigments also play a crucial role in resistance to desiccation, where water scarcity is a major limiting factor in deserts and semi-arid regions. Studies indicate that melanized fungi exhibit greater tolerance to water loss and better recovery capacity after rehydration, possibly due to the reinforced cell wall structure and the antioxidant action of melanin (Salgado-Castillo et al. 2023; Bozhuyuk and Ozdal 2025; Menichetti et al. 2025). This protection against reactive oxygen species is essential, since the combination of intense radiation and water stress favors the formation of free radicals (Li et al. 2024). Carotenoids also contribute to the adaptation of fungi to arid environments, acting mainly as antioxidants and protectors against photo-oxidative damage. This family of lipophilic pigment compounds also has the ability to help neutralize reactive oxygen species generated by UV radiation and thermal stress (Sandmann 2022; Saubenova et al. 2024). Thus, the combined presence of different pigments can enhance the adaptive capacity of fungi, allowing their persistence in extreme micro-habitats, such as biological soil crusts (Deveautour et al. 2020).

From an ecological point of view, a greater abundance of fungi with dark spores is frequently observed in increasing gradients of aridity, suggesting that pigmentation is a functional trait selected in these environments (Hopkins and Bennett 2023). Highly pigmented fungi contribute not only to their own survival but also to the stability of biological crusts, helping to protect the soil against erosion and maintain microenvironments favorable to other microorganisms. Thus, fungal pigments exert an impact that goes beyond the cellular level, directly and indirectly influencing broader ecological processes (Guang et al. 2025; Lv et al. 2025). Studies conducted in arid ecosystems have significantly expanded the taxonomic inventory of pigmentogenic fungi isolated from hot and cold deserts, hyperarid soils, semi-arid regions and biological crusts. In the semi-desert of Coahuila, Mexico, isolates of *Penicillium purpurogenum* (GH2) and *Penicillium pinophilum* (EH2 and EH3), obtained from leaves of *Quercus* spp. and *Larrea tridentata*, showed pigment production in orange-red and red-purple hues, expanding knowledge about the chromatic diversity of this genus in environments with high radiation and water limitation (Espinoza-Hernández et al. 2013).

In Asian deserts, such as the Taklamakan, the black yeast *Aureobasidium melanogenum* XJ5-1 has been described as producing dark brown intracellular melanin, associated with tolerance to multiple environmental stresses, including UV radiation, high temperatures, desiccation, acidity, high saline concentrations, and oxidative stress from H₂O₂ (Jiang et al. 2016). In the Atacama Desert region, one of the driest environments on the planet, melanized species such as *Neocatenulostroma* sp. were identified in gypsum crusts, exhibiting dark brown pigmentation attributed to the presence of intracellular melanin, whose characterization was performed by Raman spectroscopy (Culka et al. 2017). Also in the Atacama Desert, isolates of *Exophiala* sp. 15Lv1 and *Rhodosporidium toruloides* demonstrated resistance to UV radiation and the ability to produce carotenoids and melanin, reinforcing the role of these pigments in cellular protection against intense solar radiation and oxidative stress characteristic of high-altitude and extremely arid regions (Pulschen et al. 2015).

Other frequently reported genera from hot desert areas include ubiquitous fungi such as *Alternaria*, *Aspergillus*, *Cladosporium*, *Curvularia*, *Fusarium*, *Penicillium*, *Phoma* and *Talaromyces*, among others. Depending on the species, they produce diverse pigmentations, ranging from melanized to colorful, and many species of these genera have been cultivated using soils from these environments (Alsohaili and Bani-Hasan 2018; Ameen et al. 2022). This substrate, in at different sampling points in Saudi Arabia, typical dark brownish hues were obtained, namely *Alternaria alternata*, *A. chlamydosporigona*, *Aspergillus niger*, *Aspergillus phoenicis*, *Cladophialophora carrionci*, *Curvularia hawaciensis*, *Curvularia nicotiae* and *Stemphylium solani*, as well as in shades of green (*Chaetomium madrasense*, *Penicillium chrysogenum*, *Talaromyces variabliis*, *Trichoderma longibrachiatum* and *Ulocladium* sp.), yellowish (*A. oryzae* and *A. terreus*) and purplish-pink (*Fusarium brachygibbosum* and *F. oxysporium*), many of these isolates have shown capabilities in the production of various antioxidants and enzymes (Ameen et al. 2022). Similarly, isolates with typical colorations have also been reported from soils and parts of fruits in the Jordanian desert, especially grayish to dark ones (*Alternaria alternate*, *Aspergillus niger*, *Rhizopus stolonifer*), greenish (*Alternaria tenuissima*, *Alternaria gaisen*, *Penicillium citrinum*) and purple (*Fusarium oxysporum*) (Alsohaili and Bani-Hasan 2018).

In soils of the semi-arid Caatinga biome, an environment characterized by high temperatures, seasonal drought, and intense solar radiation in Brazil, isolates of *Talaromyces* sp. showed the ability to produce colored secondary metabolites with potential biotechnological applications. Three different strains de *Talaromyces* sp. (1337, 1349 and Ga0022) produced extracellular pigments with dark red, red, and yellow coloration when cultivated in media containing different carbon sources, including glucose, sucrose, fructose, maltose, and starch. The pigments showed antimicrobial activity, suggesting a protective and competitive ecological role for these compounds in soil environments (Lins et al. 2022). These pigments are commonly associated with secondary metabolites derived from polyketides produced by *Talaromyces* species, which have been extensively investigated for their potential use in biotechnology as natural dyes and antimicrobial agents (Morales-Oyervides et al. 2020). Other studies in the same biome, such as that of Silva et al. (2022a), describe several filamentous fungi that have demonstrated the ability to produce pigments, isolates identified as *Talaromyces* sp. UCP 1324, *Penicillium* sp. (UCP 01152 and UCP 1286) and *Aspergillus* sp. UCP 01349 produced orange, green, red and yellow colors when cultivated under laboratory conditions. In addition to their ecological function of adapting fungi to environmental stress factors, these fungal pigments have demonstrated potential for use in bioelectrochemical technologies (Silva et al. 2022a).

Recent records from the Eastern Province of Saudi Arabia also highlight the diversity of pigmentogenic filamentous fungi in desert soils and in the atmosphere. Species such as *Achaetomium cristalliferum*, *Aspergillus terreus*, *Chaetomium strumarium* and *Fusarium solani* showed production of extracellular pigments in shades of yellow, brown, and red, with relevant biological characterization (Alyami et al. 2025). In the study by Al-Atrash et al. (2021), which evaluated the abundance and diversity of soil yeasts in a hot semi-arid ecosystem in Baghdad, Iraq, some pigmented species were identified as yeasts *Rhodotorula laryngis*, *Rhodotorula colostri* and *Rhodosporidium babjevae* exhibited intense reddish-pink pigmentation, attributed to the production of carotenoids, compounds that perform photoprotective and antioxidant functions, reducing the effects of UV radiation and oxidative stress under conditions of high insolation and low water availability. The authors suggest that, even in arid and hot environments that impose severe challenges to microbial survival, the low frequency of pigmented yeasts may reflect both moderate selective pressure, due to sparse vegetation cover, and alternative stress tolerance strategies, such as changes in metabolism or the formation of resistant structures (Al-Atrash et al. 2021).

In an integrated manner, the taxonomic data available in recent literature indicate that the production of melanins, carotenoids and other pigmented metabolites constitutes a recurring adaptive strategy in fungi from arid ecosystems, both filamentous fungi and extremotolerant yeasts that contribute to chromatic diversity. The occurrence of these pigments is frequently associated with resistance to UV radiation, tolerance to desiccation, thermal stability, and protection against oxidative stress present in desert and arid environments (Lin and Xu 2022; Hopkins and Bennett 2024). Thus, the record of pigmentogenic species in arid regions of Mexico, Caatinga, the Taklamakan Desert, Iraq, the Atacama Desert, and the Arabian Peninsula demonstrates not only the metabolic plasticity of these microorganisms but also the biotechnological potential associated with compounds produced under extreme hot environmental conditions.

### Cold arid ecosystems

In extremely cold environments, such as polar, alpine, and cryogenic regions, the survival of fungi depends on the development of physiological and structural adaptations that allow them to withstand low temperatures, high radiation levels, limited nutrients, and desiccation conditions (Duarte et al. 2018; 2019 ; Sajjad et al. 2020). Among these adaptive strategies, the production of pigments, especially melanins, stands out. These pigments accumulate in the cell wall and confer a characteristic dark color. These pigments perform essential protective functions, acting as barriers against ultraviolet radiation, oxidative stress, and water loss, in addition to contributing to increased mechanical and chemical resistance of fungal cells (Jodłowska and Białkowska 2024). Thus, pigmentation is recognized as a fundamental multifunctional mechanism for the persistence of psychrophilic and psychrotolerant fungi in extreme niches where microbial survival is severely restricted (Sajjad et al. 2020; Jodłowska and Białkowska 2024).

In this context, melanin is an example of a pigment derived from a heterogeneous group of high molecular weight phenolic pigments, formed from the oxidation and polymerization of aromatic precursors, and widely distributed in different domains of life. In fungi, melanins are generally insoluble, electrically charged, and strongly associated with the cell wall, where they perform structural and protective functions (Strycker et al. 2021), contributing to the maintenance of cell wall integrity and energy dissipation, reducing oxidative damage under conditions of extremely limited metabolism (León et al. 2025). From a chromatic point of view, fungal pigments classified as melanins range from brown to intense black, reflecting the extensive electronic conjugation present in their polymeric structures. Among the main types of fungal melanin recognized are DHN-melanin (derived from 1,8-dihydroxynaphthalene), DOPA-melanin (originating from L-DOPA), and pyomelanin, associated with homogentisate degradation. DHN-melanin is particularly common in fungi adapted to extreme environments, a classification widely discussed in recent literature on melanin biosynthesis and functions in fungi (Jia et al. 2025).

In the Antarctic continent, considered a polyextreme environment, several microbiological surveys have recorded the frequent occurrence of melanized fungi, both filamentous and yeast-like (Duarte et al. 2019; Cavalcante et al. 2023). Among these, classic genera of black fungi stand out, such as *Cladosporium*, *Cryomyces*, *Exophiala*, *Extremus*, *Friedmanniomyces*, *Knufia* and *Nadsoniella*, widely recognized for their tolerance of extreme conditions (Sterflinger et al. 2012; Selbmann et al. 2021; Isola et al. 2022; Cavalcante et al. 2023). The genus *Cryomyces*, in particular *Cryomyces antarcticus*, has been intensively studied as a model of Antarctic endolithic life. Studies by Selbmann et al. (2005); Catanzaro et al. (2024); Pacelli et al. (2020b) demonstrate that these fungi colonize the interior of cold and extremely dry rocks, where intense melanization is a key factor for long-term survival. According to these authors, the characteristic black coloration of *Cryomyces* results from the robust production of DHN-melanin, which is densely deposited in the cell wall. This pigment not only acts as a passive chemical protector but also constitutes an essential structural component, conferring mechanical resistance and cellular stability in environments with extreme levels of radiation and minimal water availability. These characteristics make Antarctic melanized fungi excellent analogues for astrobiological studies, reinforcing the idea that melanin plays a central role in the viability of life in conditions considered limiting for the terrestrial biosphere (Selbmann et al. 2005; Pacelli et al. 2020b; Catanzaro et al. 2024).

Melanization is extremely common in dark septate endophytic fungi (DSE) isolated from cold areas, reflecting a strategic adaptation to adverse environmental conditions. This phenomenon has been recorded in several fungal genera associated with host plants in alpine and polar regions, including *Alternaria*, *Cadophora*, *Leptodophora* and *Phoma*, among others (Hou et al. 2025). These dark fungi are widely distributed ascomycetes that colonize plant roots and are characterized by melanized and septate hyphae, often organized into microsclerotia, in which melanin acts as a protective pigment, conferring dark coloration and increasing resistance to radiation, oxidative stress, and severe thermal variations. Taxonomically, DSEs form a paraphyletic group within the *Ascomycota*, encompassing taxa from the orders *Pleosporales*, *Helotiales* and *Xylariales*, and exhibit low host specificity, colonizing a wide diversity of plants (Akhtar et al. 2022; Hou et al. 2025). Symbiotic associations are considered to facilitate the resilience and survival of plants in cold ecosystems, reinforcing the ecological importance of DSEs as mediators of plant adaptation (Akhtar et al. 2022; Lopez et al. 2024; Hou et al. 2025). These associations are frequently correlated with improved water and nutrient absorption, modulation of physiological responses, and mitigation of abiotic stresses, demonstrating that melanization and DSE-plant symbiosis constitute adaptive strategies that increase the resilience of plant communities in harsh and oligotrophic environments (Acuña‑Rodríguez et al. 2024; Lopez et al. 2024).

In addition to melanized DSEs, other pigment-producing endophytes are relevant in low-temperature contexts (Andrade et al. 2023). Among the endophytic communities of Antarctic mosses (*Polytrichastrum alpinum* and *Sanionia uncinata*, pigmented filamentous fungi have been identified in genera such as *Alternaria*, *Aspergillus*, *Cladosporium*, *Diaporthe*, *Epicoccum* and *Fusarium*, demonstrating that the presence of pigmentation in the recovered endophytic strains may be associated with adaptation to this harsh environment, thereby extending the records of these taxa to the Antarctic region, although the properties of the pigments themselves were not investigated (Andrade et al. 2023).

Some fungi are commonly cited as prolific producers of high-quality red pigments at low temperatures, although not fully characterized structurally, including the proposal of new lineages such as the species *Victoriomyces antarcticus* (Davolos et al. 2019) and others with efficiency in producing this color, among them *Geomyces* and/or *Pseudogymnoascus* are major highlights of low-temperature habitats (Long et al. 2024; Zhou et al. 2025). This latter genus is quite common in these ecosystems, and some lineages exhibit very characteristic pigmentation (Duarte et al. 2019), with the production of various reported bioactive natural secondary metabolites (Fujita et al. 2021; Antipova et al. 2023). The coloration of the colonies can vary depending on the species, the culture medium and the incubation temperature. In Antarctica, four new species of this genus were proposed by Villanueva et al. (2021), with *P. antarcticus* and *P. lanuginosus* exhibiting extracellular pigments in shades of yellow and cinnamon, respectively, when grown on Sabouraud Agar (SBA) at 15 °C. While other strains produce the typical reddish-wines, possibly azaphilones as recently proposed (Palma et al. 2024). Cavalcante et al. (2024) demonstrated that Antarctic isolates of *Pseudogymnoascus* spp. produce extracellular pigments, with predominantly pink to violet colors when cultivated on solid media, whose extracts showed antimicrobial and antiparasitic bioactivities. Pigmentation in this fungus also appears to be associated with adaptive responses to environmental stresses, Wong et al. (2022) showed that some isolates of this strain exhibited darkening of their colonies in response to UV-B radiation, sometimes with reddish or brown tones, indicating possible modulation of the production of pigments such as melanin or other phenolic compounds.

In response to low temperatures, other important filamentous fungi adapted with pigmentation are present, including species of *Antarctomyces*, *Penicillium*, *Thelebolus* and *Talaromyces*, for example. Pandey et al. (2018) described a psychrotolerant strain of *Penicillium* sp. (GBPI_P155) isolated from high-altitude soils in the Himalayan region, which produced an orange pigment. Chemical characterization of the pigment indicated that it is composed of carotenoid derivatives, specifically tangeraxanthin and 4-ketonostoxanthin, representing one of the first records of carotenoid pigments produced by cold-adapted Himalayan fungi. Although pigment production was confirmed and chemically defined, the study did not specify whether the pigment is intracellular or extracellular, nor did it evaluate associated biological activities, serving primarily as an example of chemical adaptation of fungi to low-temperature environments. Nonzom and Sumbali (2021) expanded the understanding of the occurrence of pigmented fungi in cold environments by investigating fungal diversity in high-altitude, low-temperature soils of the Drass Valley in the Indian Himalayas. The study revealed the predominance of species of the *Penicillium* genus, many of which presented visibly pigmented colonies, with typical colors ranging from bluish-green, blue-green, and grayish tones, recurrent characteristics of the genus. Although the authors did not perform chemical characterization of the pigments produced, the wide distribution of *Penicillium* suggests that pigment production is a common phenotypic trait among adapted species, and may be associated with tolerance to environmental stresses characteristic of cold region (Nonzom and Sumbali 2021).

In the fungus *Thelebolus microsporus*, isolado das Larsemann Hills (Antártica), isolated from the Larsemann Hills (Antarctica), HPLC analysis revealed the presence of a yellow carotenoid pigment, suggesting that its production may be associated with adaptation to extreme cold (Singh et al. 2013). The strain presented polyunsaturated fatty acids, such as linolenic acid, and activity of extracellular enzymes, such as α-amylase, with potential industrial applications. Also from the Antarctic polar area, the species *Talaromyces cnidii* produced an extracellular pigment of pink to violet color, whose extract exhibited antimicrobial activity against *Staphylococcus aureus*, highlighting the biotechnological potential of fungal pigments from polar environments (Cavalcante et al. 2024). The species *Antarctomyces pellizariae*, described by de Menezes et al. (2017) as a new psychrophilic ascomycete from Antarctic snow, exhibits a remarkable blue coloration. The pigment responsible has not been chemically characterized, but the authors highlight that this consistent hue may be related to protection against radiation or oxidative stress, characteristics common to fungi adapted to icy and highly illuminated environments.

In turn, yeasts associated with cold environments constitute an ecologically relevant group, frequently dominant in fungal communities of polar, subpolar and cryogenic regions, exhibiting high taxonomic and functional diversity (Buzzini et al. 2017, 2018; Duarte et al. 2018; Silva et al. 2022b). These microorganisms exhibit a set of physiological adaptations that favor their survival and metabolic activity at low temperatures, including alterations in the composition of cell membranes, production of cryoprotective compounds, and synthesis of cold-active enzymes (Duarte et al. 2018; Silva et al. 2022b). Pigmented yeast taxa are frequently recorded in cold habitats, exhibiting intense production of photoprotective pigments that give them characteristic coloration and greater tolerance to abiotic stressors such as UV radiation, freezing, and oxidative stress (Kreusch and Duarte 2021). These organisms belong mainly to the phylum *Basidiomycota* (Buzzini et al. 2018; Silva et al. 2022b), although ascomycete representatives are also recurrent in these extreme environments (Moliné et al. 2013; Selbmann et al. 2014).

In previous observations of pigmented strains isolated from polar and alpine environments, colorations ranging from orange and reddish to pinkish were frequently found, chemically characterized as carotenoids (Vaz et al. 2011; Feng et al. 2025), with *Rhodotorula* spp. being one of the best-represented genera (Cavalcante et al. 2023). For example, the species *R. frigidialcoholis*, analyzed by Touchette et al. (2021) from samples of Antarctic permafrost, produces carotenoids typical of this basidiomycete genus, such as torularodin and β-carotene, responsible for its characteristic reddish-orange coloration. These pigments act as antioxidants and radiation protectors, playing a relevant role in survival under glacial and subglacial conditions. Although the study’s focus was not specifically on pigments, the authors highlight the importance of carotenoids in cold and radiation tolerance (Touchette et al. 2021). Similarly, Singh et al. (2014) reported *R. svalbardensis* also orange in color and producing β-carotene and torularhodin, isolated from glacial soil and marine sediment samples from Svalbard Island.

Moving beyond predominantly phenotypic characterization, Amaretti et al. (2014) investigated psychrotolerant yeasts isolated from the ice surface of an alpine glacier in northern Italy. Among the isolates, three strains with pigmented colonies were identified by ITS sequencing as belonging to *R. mucilaginosa*, *R. laryngis* and *Dioszegia* sp. The colonies exhibited colors ranging from pink to orange, associated with the presence of carotenoids, confirmed by UV-Vis spectrophotometry and HPLC-DAD; in *Dioszegia* sp., the analysis indicated a compound compatible with dihydroxylated xanthophylls. The authors suggest that their production has an adaptive function, protecting the cells against UV radiation, extreme cold, and oxidative stress, in addition to aiding in the phenotypic differentiation and taxonomic classification of glacial yeasts, reinforcing the potential of pigmented fungi from supraglacial environments as sources of carotenoids adapted to low temperatures (Amaretti et al. 2014).

Other pigmented yeast-like basidiomycetes already found in these cold areas belong to the *Rhodosporidiobolus* and *Sporobolomyces* (Duarte et al. 2019). Isolated from glacial ice, the species *Rhodosporidiobolus oreadorum* has been reported as having pink to reddish colonies, with carotenoid production associated with adaptation to low temperatures and UV radiation (Turchetti et al. 2018). Also exhibiting pink coloration, *Sporobolomyces roseus*, recently isolated from Livingston Island (Antarctica), has recently been described as a promising source of cold-environment carotenoids and studied for its metabolic profile and biotechnological potential (Davoli and Webe 2002; Rusinova-Videva et al. 2024). In glacial habitats of Greenland and Svalbard, Perini et al. (2021) described the colorful taxa of the genus *Camptobasidium* with *C*. *gelus*, the proposed new species *C. arcticum* and the dimorphic fungus *Psychromyces glacialis*. The species showed growth at low temperatures, evidencing psychrotolerant physiological adaptations compatible with glacial ecosystems. In culture, the colonies exhibited coloration ranging from pale pink to reddish, a recurring phenotypic characteristic among the isolates. Although pigmentation was not used as an isolated taxonomic criterion, it represents a relevant morphological trait, potentially related to cellular protection against stresses typical of cold environments. Thus, as visualized here, cold desert areas harbor an important profile of mycelial pigments and yeasts from different environmental sources, actively exhibiting melanins, carotenoids, and azaphilones, thereby demonstrating great taxonomic relevance and potential.

### The genome of adapted and pigmented fungal lineages from arid and cryospheric habitats

Advances in microbial genomics have enhanced studies of fungal biology in relation to the content of their genomes, with increasing attention paid to taxa derived from arid and cold desert niches. The goal is to understand the adaptive and biosynthetic events present in these eukaryotes, as well as their lifestyles, evolutionary aspects and useful ecological and biotechnological functionalities (Knapp et al. 2018; Wei et al. 2020; Zajc et al. 2022; Shin et al. 2025; Silva et al. 2025b). This represents a growing field of research with future relevance and has inspired projects directed towards this mission with extremophiles (Hilário et al. 2019; Selbmann et al. 2020). The genomes of pigmented fungi can reveal details of their extreme natures and cellular commands, including the gene content linked to the synthesis of their colored components and other useful directions of their adaptations (Sterflinger et al. 2014; Wei et al. 2023; Mi et al. 2024).

In the genome of ascomycete fungi of the genera *Aspergillus*, *Coniochaeta*, *Embellisia*, *Chaetomium* and *Phoma*, exhibiting them as dark in culture, obtained from soils of an arid grassland terrestrial ecosystem near Moab, Utah, USA, Challacombe et al. (2019) observed genes predicted for melanization, with evidence for the biosynthesis of three types of melanin: DHN melanin (derived from dihydroxynaphthalene compounds), eumelanin (via the L-3,4-dihydroxyphenylalanine L-DOPA pathway), and pyomelanin (degradation of L-tyrosine), with genetic compatibility for the genes of polyketide synthases (PKS), tyrosinases, and 4-hydroxyphenylpyruvate dioxygenase (HPPD). These dark brownish pigments accumulated in the cell wall are indicated as good strategies for the abiotic environmental resistance of these fungi in arid areas and are also linked to pathogenicity in ecological interactions with plants and animals (Challacombe et al. 2019). In the yeast-like fungi *Aureobasidium melanogenum* (XJ5-1) from the Taklimakan Desert, the cell wall integrity (CWI) signaling pathway, via the MAPK Slt2 and transcriptional activator Swi4, has been shown to play a central role in the biosynthetic regulation of DHN-melanin through modulation of the transcriptional factor Cmr1 (CMR1 gene), which has an active effect on the expression of genes for these pigments such as PKS1 in this strain (Jiang et al. 2020). Based on the genomic information of this fungus, in addition to melanin synthesis, trehalose accumulation mediated by the function of the TPS1 gene (trehalose-6-phosphate synthase) also contributes to the environmental versatility of this desert-derived fungus in the face of stressors such as heat shock, high oxidation and desiccation (Jiang et al. 2018).

This accumulation of melanin in fungal cells from extreme habitats is a significant characteristic also noted in polar lineages, for example, in the draft genome of the strongly melanized endemic strain of *Cryomyces antarcticus* (CCFEE 534), recovered from the McMurdo Dry Valleys of the cold Antarctic desert (Selbmann et al. 2005), the genes for laccase and PKS, linked to the synthesis of this dark pigment, were present, in smaller quantities than in other comparative melanized fungi, but identified as genetically conserved genes among clonal individuals of this fungus, directly linked to its survival capacity and environmental polyextremophily (Sterflinger et al. 2014). Recently, in the genome of the CCFEE 515 genotype of this Antarctic fungal genus, DHN melanin was identified as the prevalent type of melanin pigment in this fungus. This was due to the identification of putative genes involved in its melanogenesis and polymerization, notably *capks1*, which encodes a non-reducing polyketide synthase, CaPKS1, that provides precursors for DHN melanin in *C*. *antarcticus*. This was confirmed after knocking out this gene, which prevented melanization in *Δcapks1* mutants. The coding region of *Δcapks1* was deleted from the fungal genome using a CRISPR/Cas9-assisted replacement approach. Furthermore, this deletion caused structural changes in the organization of the cell walls of the knockouts, thus highlighting this gene as a key element in the synthesis and function of DHN melaninin this extreme representative (Catanzaro et al. 2024).

In a comparative genomic approach, melanized *C*. *antarcticus* (isolate 116301) was used with other dark fungi, including psychrophilic representatives and those with varied lifestyles (pathogens, lichenized, and other extremophiles [acidophilic, heat- and salt-tolerant]), aiming to elucidate adaptations associated with extreme lifestyles, Gomez-Gutierrrez et al. (2024) reported a number of interesting characteristics for the genomic cluster formed by psychrophiles, observing a high content of CG, along with the expansion of certain gene orthogroups linked to genes predicted for enzymes involved in various cellular functions, related to energy metabolism and modulation of cell wall integrity (CAZymes, alcohol dehydrogenases), transporters (major facilitator superfamily - MFS), as well as in the stabilization of cellular genetic material, combating oxidative damage by reactive oxygen species and melanin biosynthesis (helicases, Zn(2)-C6 type transcription factors and proteins with G-patch domain). On the other hand, some gene families were found to be reduced in psychrophilic fungi, including *Cryomyces*, mainly related to biological interaction processes for immune defense, competition, and pathogenicity with other organisms in the natural environment. For example, contraction was observed in the effector gene Ecp2, present in described pathogen-host interactions in phytopathogenic fungi. These findings in the genomes of the inspected cryospheric fungal species are justified by the restrictive environmental isolation they face, and the main investment seems to be directed towards assembling adaptive responses to the abiotic conditions prevalent in the cold areas where they occur (Gomez-Gutierrrez et al. 2024).

Efforts have been made to describe the molecular basis of metabolites in pigmented fungi adapted to cold areas, especially seeking to identify the specific biosynthetic gene clusters (BGCs) and differentially expressed genes (DEGs) responsible for their typical colors or bioactivities from their genomes (Batista et al. 2020; Long et al. 2024; Palma et al. 2024). Producing a diffusible reddish pigment in pure culture, the genomic screening of the strain *Pseudogymnoascus verrucosus* (FAE27), isolated from an Antarctic sponge, it was revealed to contain a putative cluster of azaphilone biosynthesis genes, named *azp* BGC, possibly involved in the synthesis of the red pigment in this fungus. This cluster encompasses a group of twelve genes with distinct predicted functions. Among them, through the use of RNA interference-mediated gene silencing (RNAi) and functional disruption by CRISPR/Cas9, along with chemical analysis by HPLC (peaks at 530 nm), the azpA gene in this BCG, encoding a non-reducing polyketide synthase (NR-PKS), was confirmed as the determinant in the expression of metabolites responsible for the red coloration of the azaphilone type in the investigated strain (Palma et al. 2024). The red pigmented psychrotrophic fungus *Geomyces* sp. WNF-15 has also been the focus of comparative genomic investigations between wild-type strains and mutants generated by gene editing using omics techniques. This allowed the identification of eleven differentially expressed genes in the regulation of the synthesis of these metabolites and influencing production at higher temperatures than the standard psychrophilic condition (at 14 °C), with a direct effect on optimization when scaffold1.692 (Δ1-692) and scaffold2.t704 (Δ2-704) were absent (knockout) (Long et al. 2024).

The sequenced genome of the blue fungus endemic to the Antarctic environment, *Antarctomyces pellizariae*, showed BCGs linked to the production of putative genes encoding an ice-binding protein, possibly related to adaptation to low temperatures. However, the precise characterization of the biosynthetic pathway of its blue pigment, one of the rarest colors ever seen in nature and also among described fungi, remains unknown to date (Batista et al. 2020). Much of the genomic understanding of colored fungal lineages from arid areas needs further exploration, which could contribute to the concrete industrial application of these fungi, gene sequences and their cellular products.

## Biotechnological reasons of fungal biopigments from arid and cryospheric habitats: Prospecting and application potential

### Environmental screening for extremophilic pigmented fungal strains

Growing interest in natural pigments has driven the search for new biological sources capable of meeting industrial demand for safer, sustainable, and functionally active coloring compounds. In this context, microbial sources stand out as promising alternatives to pigments of plant or synthetic origin. Overall, microorganisms offer the advantage of faster growth and shorter production cycles, while being independent of seasonal or climatic factors, thereby enabling continuous, scalable production throughout the year (Kalra et al. 2020). Furthermore, ease of cultivation in controlled fermentation systems allows for greater standardization, reproducibility, and optimization of pigment yields—including through the adjustment of physicochemical parameters and the use of low-cost substrates such as agro-industrial waste—reinforcing the sustainable and economically viable nature of these production systems (Kalra et al. 2020; Chavan et al. 2025). In this context, fungi isolated from extreme or little-explored environments, such as polar regions, deserts, semi-arid areas, and oligotrophic substrates, and have stood out as promising sources of structurally diverse secondary metabolites (Duarte et al. 2019; Menezes et al. 2023). The adverse environmental conditions of these niches can drive the selection of eukaryotes adapted with specialized metabolisms, often associated with the production of pigments with multiple functions (photoprotection, resistance to oxidative stress, and thermal adaptation) (Cavalcante et al. 2023).

**Fig. 3:**
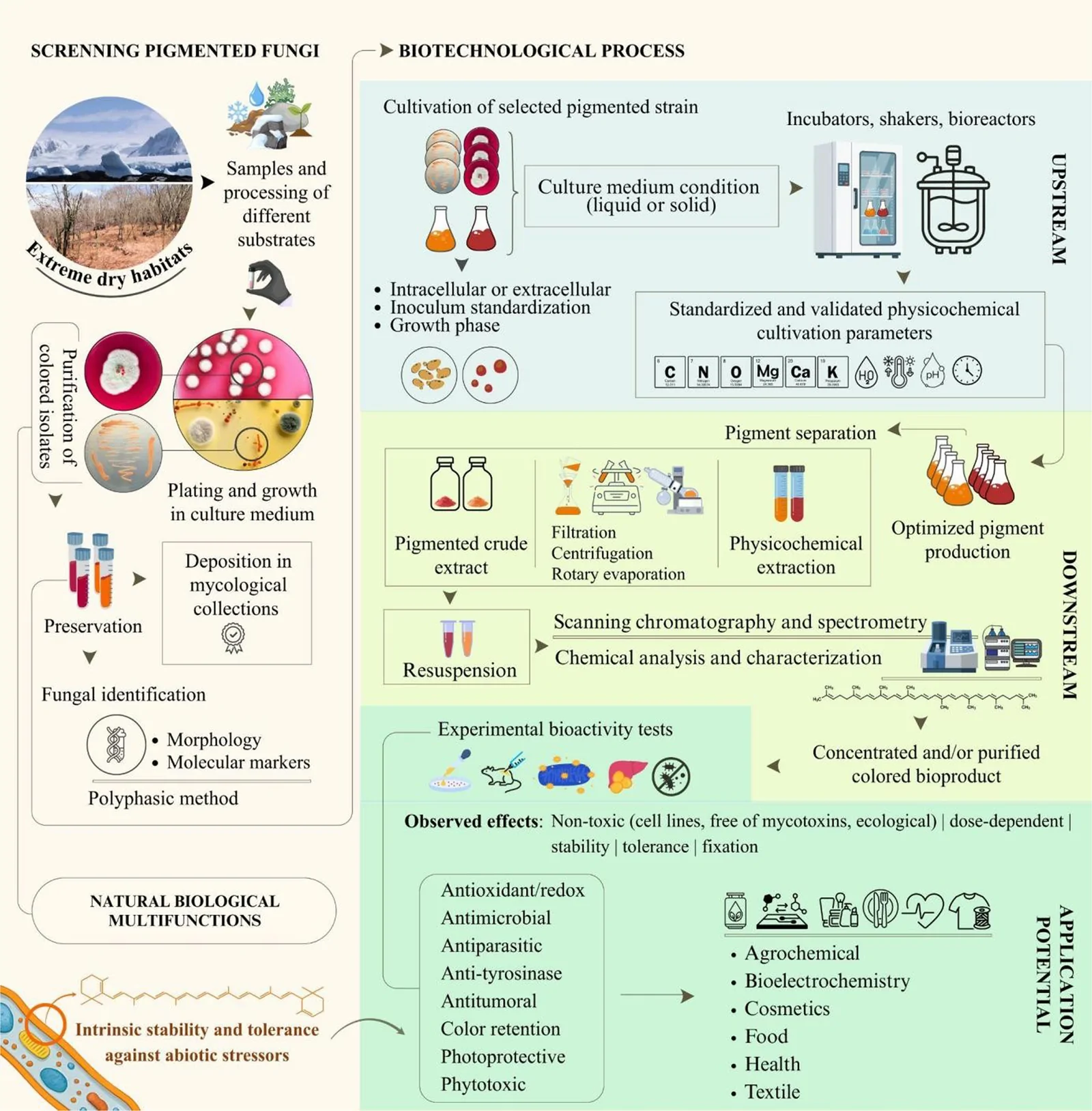
Diagram of the bioprospecting pathway for pigmented fungi, from environmental screening to biotechnological stages and applied industrial directions for their metabolites

Screening (bioprospecting) of pigmented fungi is the initial and fundamental step in the biopigment development workflow, responsible for identifying strains with relevant biosynthetic capacity (Fig. 3). This phase guides the subsequent stages of cultivation, production, extraction, and structural characterization of the pigments, in addition to contributing to the optimization of laboratory resources (Duarte et al. 2019). The careful selection of strains allows efforts to be directed towards microorganisms with greater biotechnological potential, considering parameters such as intensity and stability of coloration, chromatic diversity, intra- or extracellular pigment profile, and viability of cultivation on a laboratory scale (Olicón-Hernández et al. 2022; Rosa et al. 2022).

This process generally begins with environmental screening, in which samples are collected from different terrestrial, aquatic, marine, and/or atmospheric natural substrates/hosts of the extreme ecosystems of interest. After collection under aseptic conditions, the fungi are recovered by classical cultivation methods in appropriate solid media, e.g., Malt extract agar (MEA), Potato dextrose agar (PDA), Sabouraud agar (SAB) and Semi-Synthetic Mineral Medium (MMS), followed by successive purification steps to obtain pures cultures (Germain et al. 2021; Huang et al. 2022; Černoša et al. 2024). The safe and aseptic *ex situ* preservation of these pure extreme lineages with laboratory pigmentation is essential to ensure their genetic and phenotypic stability, allowing for reproducible analyses and the precise study of their metabolites and potential subsequent biotechnological applications, as well as their taxonomic positions, which is important for the consolidation of robust and representative mycological collections (Danner et al. 2023). Many methods are employed for this purpose, encompassing short- to long-term conservation techniques, including immersion in distilled water (Castellani 1967), mineral oil, lyophilization, liquid nitrogen, and/or ultra-freezing in cryoprotective solution (glycerol) (Talib et al. 2022).

After selecting and preserving colored fungi in culture, systematic identification is performed based on the polyphasic method, which combines morphological characteristics of the cultures and phylogeny of nuclear and mitochondrial multigenic biomarkers such as ribosomal genes (small/large subunits and Internal Transcribed Spacer), β-tubulin (benA, tub-2), translation elongation factor 1-α (TEF), calmodulin (caM), cytochrome b (Cyt b) and RNA Polymerase (RPB 1 and 2) (Gannibal 2022). Once bioprospected and properly isolated from their extreme environments, fungal strains are cultivated under standardized laboratory conditions in solid or liquid media, allowing for macroscopic evaluation of growth and pigment production (Souza et al. 2016). The presence of intense colors, whether diffusely in the medium (extracellular) or associated with cellular structures (endocellular), constitutes a preliminary indication of the production of pigmented metabolites and allows for the rapid identification of promising isolates. In addition to visual evaluation, screening may involve comparing performance in pigment production and intensity under different cultivation conditions, such as variations in pH, temperature, carbon and nitrogen sources, aeration and incubation time (Pandey et al. 2018; Toma et al. 2023; Pereira et al. 2024). These approaches allow us to assess the metabolic plasticity of strains and identify conditions that favor or suppress the biosynthesis of their pigments, an aspect that is particularly relevant since the production of secondary metabolites is closely related to the physiological state of the microorganism and the environmental conditions imposed (Lin and Xu 2022).

In this scenario, environmental screening in understudied desert areas acts as an indispensable strategic filter in the process of developing natural fungal pigments, reducing the intense complexity of these environments by applying cultivation methods to their different sampled sources and allowing the recovery and selection of the most promising strains to advance to further research (Heo et al. 2018; Lu et al. 2023). This process is especially relevant in the context of extremophile fungal bioprospecting, where the metabolic diversity associated with environmental adaptation represents a valuable opportunity for the discovery of beneficial pigments with more efficient and eco-compatible industrial, pharmaceutical, and environmental applications (Cavalcante et al. 2023).

### Upstream and downstream processing of extremophilic fungal pigments

Once fungal strains with pigmentation in active growth have been recovered through initial screening, it becomes necessary to conduct studies focused on consolidating cultivation and production conditions, a set of actions that mark the upstream stage of the process (Fig. 3). This phase encompasses the set of strategies employed to maximize pigment biosynthesis during microbial cultivation, including inoculum standardization, culture medium formulation, and control of the physicochemical variables of the fermentation system. In the context of fungal pigment production, the upstream stage plays a central role, since the synthesis of these compounds is closely associated with secondary metabolism and, therefore, highly dependent on the environmental conditions imposed on the microorganism (Valkenburg et al. 2024).

Changes in the nutritional composition of the medium, oxygen availability (aeration), pH, temperature, and cultivation regime can result in significant variations in both the yield and the chemical and chromatic profile of the pigments produced (Suwannarach et al. 2019; Venkatachalam et al. 2021, 2023). Therefore, understanding the factors that regulate fungal pigmentogenesis is essential for the development of reproducible and scalable processes (Alves et al. 2025). Inoculum preparation (number of cells/conidia per volume) is one of the first critical upstream steps, directly influencing the growth kinetics and the metabolic phase in which pigment production occurs (Morales-Oyervides et al. 2015; Ruiz-Sánchez et al. 2023), which fungi generally occurs during the activation of secondary metabolism in the stationary growth phase. Aspects such as physiological age, initial biomass concentration, and fungal morphological form must be carefully controlled, as they can impact the efficiency of the production process. Standardized inocula tend to reduce experimental variations and favor comparability between different cultivation conditions (Morales-Oyervides et al. 2017; Veiter et al. 2018).

The formulation of the culture medium is another determining factor for the induction of pigment production. Sources of carbon, nitrogen, and other elements, as well as their proportions, modulate the activation of specific biosynthetic pathways, and can stimulate or repress the production of secondary metabolites (Molelekoa et al. 2021). Furthermore, supplementation with mineral salts, metabolic precursors, or agro-industrial residues has been widely explored as a strategy to increase pigment yield and simultaneously reduce process costs, aligning with the principles of sustainability and circular economy (Lopes and Ligabue-Braun 2021; Venkatachalam et al. 2021; Campanhol et al. 2023). The physicochemical conditions of the culture, such as pH and temperature, also exert a direct influence on the metabolic expression of pigment-producing fungi. In many cases, pigmentation is intensified under controlled stress conditions, reflecting the adaptive role of these compounds in the face of environmental variations (Huarte-Bonnet et al. 2025). Additionally, parameters such as agitation and aeration affect the transfer of oxygen and nutrients, influencing fungal morphology and, consequently, the efficiency of the fermentation process (Veiter et al. 2018). Cultivation in bioreactors also helps in controlling and defining the ideal physical-chemical parameters to favor the production of pigments by the selected strains (Valenzuela-Gloria et al. 2021).

The cultivation regime adopted, whether in a submerged system (broth), surface cultivation (solid), or semi-solid, should be selected according to the characteristics of the strain and the pigment of interest (Duarte et al. 2019). While submerged cultures favor scalability and control of operational parameters, solid-state cultures can stimulate the production of certain pigments associated with the air-medium interface. The choice of fermentation system, therefore, represents a strategic upstream step, directly impacting subsequent downstream steps (Ramesh et al. 2022; Rengifo et al. 2022; Qin et al. 2023). Thus, the upstream process establishes the basis for the overall efficiency of the bioprocess, determining not only the quantity of pigment produced, but also its quality, stability, and recovery viability. Optimizing this step is essential for the advancement of pigmented fungi, especially those originating from extreme environments, from the exploratory stage to biotechnological applications on a laboratory and, potentially, industrial scale (Gautério et al. 2023; Long et al. 2024).

In addition to the traditional optimization of nutritional and physicochemical parameters, the One Strain Many Compounds (OSMAC) approach has established itself as a valuable strategy for expanding the chemical and chromatic diversity that can be obtained from a single fungal isolate. This concept is based on the observation that fungal genomes harbor a repertoire of biosynthetic gene clusters considerably larger than that expressed under standard laboratory conditions, with many of them remaining transcriptionally silent in the absence of specific environmental stimuli (Bode et al. 2002; Pan et al. 2019; de Andrade et al. 2025). By systematically varying the composition of the culture medium, the carbon and nitrogen sources, the pH, the temperature, aeration, and salinity—or by supplementing the medium with chemical elicitors and epigenetic modulators—it becomes possible to activate cryptic biosynthetic pathways and thus access pigments or pigment variants that would otherwise not be produced (Pan et al. 2019). In the context of extremophilic and extremotolerant fungi, this strategy is particularly relevant, since strains adapted to nutritionally poor or physically stressful environments may retain a broad and still largely unexplored biosynthetic potential, which only becomes evident when culture conditions simulate—or even exceed— the stress levels encountered in their natural habitat (Chávez et al. 2015; Romano et al. 2018).

A complementary strategy for inducing the expression of silenced biosynthetic pathways is the simultaneous cultivation of two or more microbial strains in the same system, an approach known as co-culture. The rationale behind this strategy lies in the ecological interactions established between the co-cultured organisms, including competition for nutrients and space, interspecies quorum sensing, and chemical cross-talk, which can trigger defensive or competitive metabolic responses not observed in pure cultures (Netzker et al. 2015; Selegato and Castro-Gamboa 2023). In fungal systems, co-cultivation has been reported to enhance pigment production and, in some cases, to induce the synthesis of novel metabolites absent in both monocultures, reflecting genuine biosynthetic activation rather than a simple additive effect of the two chemical profiles (Liu et al. 2024; Wu et al. 2024).

Once a promising cultivation condition has been identified—whether derived from OSMAC screening, co-cultivation, or conventional univariate experiments—Response Surface Methodology (RSM) provides a statistically robust framework for the fine-tuning of pigment yield. Unlike the traditional approach of varying One Factor at a Time (OFAT), RSM allows multiple independent variables—such as pH, temperature, incubation time, and substrate concentration—to be evaluated simultaneously, capturing not only their individual effects but also their interactions on the response of interest (Breig and Luti 2021; Saber et al. 2023; Chalakkara and Thomas 2025). The optimization process generally begins with a screening design, such as the Plackett-Burman design, to identify the variables with the most significant influence on pigment production, followed by the application of a Central Composite Design (CCD) or a Box-Behnken Design (BBD) to construct a second-order polynomial model describing the relationship between the selected variables and the response (Zhou et al. 2009; Ghazanfari et al. 2025). The fit of the resulting model is evaluated using analysis of variance (ANOVA), considering parameters such as the coefficient of determination (R²) and the significance of the lack of fit, while three-dimensional response surface and contour plots allow visualization of the predicted optimal point (Chen and Chen 2025). Experimental validation of the predicted conditions is subsequently performed to confirm the model’s reliability. When applied following OSMAC screening or co-cultivation, RSM substantially reduces the number of experiments required to achieve maximum pigment yield, while providing a mathematically grounded prediction of the global optimum of the fermentation system (Romano et al. 2018; Zheng et al. 2025).

In the biotechnological process, after the best cultivation conditions have been established and optimized upstream, the implementation of efficient downstream strategies becomes essential (Fig. 3). This includes the set of steps aimed at the recovery, concentration, purification, and stabilization of fungal pigments, and is crucial for the technical, economic, and regulatory viability of these bioproducts (Rodríguez-Durán et al. 2011; Meruvu and Santos 2021; Cavalcante et al. 2023). In microbial systems, particularly fungal ones, this phase is one of the most expensive and crucial steps in the production process, making the careful selection of strategies compatible with the chemical nature of the pigment, its cellular location, and the intended final application essential (Khatami et al. 2026).

For this step, the pigment is initially separated from the fungal biomass in the fermentation medium by centrifugation or filtration, depending on the cultivation regime (submerged or surface), the morphology of the isolate (mycelial or yeast-like) (Iram et al. 2022), and whether production is endocellular or extracellular (diffusible in the medium). Extracellular pigments, often water-soluble, can be more easily recovered directly from the cell supernatant, significantly reducing downstream complexity (Krakauskaitė et al. 2025). This production can also be carried out using mycelium immobilized on supports in fermentation broths (cotton and nylon sponges and stainless-steel sponges, for example) (Alyami et al. 2025). In contrast, intracellular pigments, such as carotenoids produced by yeasts and filamentous fungi, require additional cell disruption steps to release the compound of interest from other components (Nemer et al. 2021; Zhao et al. 2024). Cell lysis can be conducted using physical, chemical, or mechanical methods, or even a combination thereof, with the use of abrasion with glass microspheres, homogenization and sonication widely reported as effective for disrupting the fungal cell wall. The choice of method should consider the structural integrity of the pigment, since excessive forces or extreme conditions can promote oxidative degradation or molecular isomerization (Flieger et al. 2018; Elmaaty et al. 2022; Gautério et al. 2023).

For the separation of pigments from cellular biomass, solvents are generally used to facilitate the extraction of these molecules. The polarity of these solvents must be compatible with the chemical class or set of compounds of interest. Water, acetone, alcohols (ethanol, methanol), ethyl acetate and ethyl ether are classic solvents used for this purpose. Studies demonstrate that mixtures of polar and non-polar solvents, such as acetone: hexane or acetone: ethyl ether, show high efficiency in the recovery of carotenoids, simultaneously favoring pigment solubilization and the removal of lipid impurities. For polar or water-soluble pigments, such as certain azaphilones and polyketides, aqueous or alcoholic systems can be employed, reducing the environmental impact of the process (Gmoser et al. 2017; Lebeau et al. 2017, 2020; Kanno et al. 2021; Naz et al. 2023). Subsequently, the cellular biomass of the studied fungus is separated from the solution of interest (solvent + metabolites) by phase filtration, with the solvents removed by evaporation under reduced pressure, usually in a rotary evaporator, to generate a crude extract that concentrates the pigments, which is dried using a vacuum concentrator. The resulting extract is then diluted in water (H₂O), DMSO (Dimethylsulphoxide (CH₃)₂SO), methanol (MeOH), acetone (C₃H₆O) or by using other diluting solvents. Alternatively, more environmentally friendly, solvent-free techniques can be applied to the extraction of fungal pigments, including ultrasound, pressurized liquid, microwaves and pulsed electric field (Kalra et al. 2020). All these steps must be carried out under controlled temperature and light-free conditions to avoid pigment degradation (Kumar et al. 2023).

The fractionation step (and dereplication process) is important to increase pigment purity and eliminate co-extracted interfering compounds, such as lipids, proteins, polysaccharides, and cellular debris. To this end, chromatographic techniques are widely used, notably thin-layer chromatography (TLC), column chromatography, and high-performance liquid chromatography (HPLC), applicable to both qualitative and quantitative analyses as well as preparative procedures (Gmoser et al. 2017; Mendonça et al. 2021; Zhou et al. 2023). The efficiency of purification is directly related to the appropriate selection of the stationary phase, frequently silica gel-based, as well as the composition of the mobile phase; these parameters determine the selectivity, resolution, and yield of the process, and are commonly adjusted according to the chemical characteristics and polarity of the pigment of interest (Mahato et al. 2019; Nemer et al. 2023).

The pigment concentrated and/or fractionated the mixture of dry and diluted extract can then be subjected to structural characterization using spectroscopic and/or chromatographic techniques such as ultraviolet-visible spectrophotometry (UV-Vis), Fourier transform infrared spectroscopy (FT-IR), high-performance liquid chromatography (HPLC), liquid chromatography coupled to mass spectrometry (LC-MS), and nuclear magnetic resonance (NMR), which are fundamental to confirming molecular identity and meeting regulatory requirements (Valenzuela-Gloria et al. 2021; Maia et al. 2025). These are diverse techniques with advantages and limitations that can be combined to improve this elucidation (Maia et al. 2025). Information generated by microscopy such as scanning electron microscopy (SEM), transmission electron microscopy (TEM), or atomic force microscopy (AFM) also assists in elucidating the ultrastructures of the pigments studied (Tang et al. 2025).

In the journey through the bioprospecting field of extremophilic fungi, the isolation and purification of promising strains for producing active staining during cell growth generally sparks interest in molecularly characterizing this typical color. Furthermore, testing the potential of these molecules, revealing diverse and powerful bioactive properties (photoprotective, antioxidant, and antimicrobial effects, for example), enhances this characterization using known techniques, also opening perspectives for approaches at the genetic (e.g., CRISPR/Cas9), genomic (biosynthetic pathways) and metabolomic levels (Kalra et al. 2020; Palma et al. 2024). Depending on the objective of the potential investigation, it is crucial to test the safety and stability of the biopigment, using methods that assess its possible toxic effects (cyto-, geno-, and eco-toxicity) to determine whether or not to continue research for a concrete biotechnological use (Molelekoa et al. 2023; Alyami et al. 2025).

Pigment stability is also a critical downstream step in maintaining bioactivity, especially when the objective is application in food, cosmetics, or pharmaceuticals (Valenzuela-Gloria et al. 2021; Tang et al. 2025). Strategies such as encapsulation (nanomaterials), pH adjustment, addition of natural antioxidants, and storage under an inert atmosphere have been described as effective in prolonging the stability, shelf life, and active properties of the final product (Contreras-Machuca et al. 2022; Divya et al. 2025). In general, the downstream of fungal pigments should be conceived as an integrated and scalable process, prioritizing recovery efficiency, reduction of the use of toxic solvents, and compliance with green chemistry principles. Simplifying the steps, especially for extracellular pigments, coupled with the use of selective purification methods, represents one of the main challenges and, simultaneously, one of the greatest opportunities for the industrial consolidation of these fungal biopigments, particularly those derived from extremo-tolerant strains from understudied dry environments (Cavalcante et al. 2024).

### Potential for industrial application

Cultivable colored fungi isolated from arid environments, both cold and hot, may have a wide spectrum of potential applications in different branches of biotechnology, due to their specialized metabolisms that lead to the production of different bioactive metabolites with diverse applications. Here, we bring together both the potential of pigmented strains reported from these areas and their general metabolism (i.e., when applied directly for some purpose), as well as studies focused on their pigments, highlighting their potential in agrochemical, cosmetic, food, pharmacological/health, textile and other diverse sectors (Fig. 3; Table 1).

**Table 1:** Biotechnological potential of pigmented fungi from extreme desert environments in different application segments

Fungi	Color pigment	Type pigment	Application potential (sector)	Reference
*Achaetomium cristalliferum*	Red	Not defined	Food Colorant; Textile dyeing	Alyami et al. (2025)
*Aspergillus* sp.	Red	Not defined	Bioelectrochemical	Silva et al. (2022a)
*Aureobasidium subglaciale*	Black	Melanin	Biological control against phytopathogens (agrochemical)	Zajc et al. (2022)
*Cladosporium* sp.	Black	Melanin	Phosphate solubilization (agrochemical)	Silva et al. (2025b)
*Chaetomium globosum*	Yellow	Azaphilone analogue	Potentially phytotoxic agent (agrochemical)	Zhang et al. (2021)
*Chaetomium strumarium*	Reddish-brown	Not defined	Food Colorant; Textile dyeing	Alyami et al. (2025)
*Comoclathris* sp.	Black	Melanin	Tyrosinase inhibitory (Cosmetic)	Georgousaki et al. (2022)
*Cryomyces Antarcticus*	Black	Melanin	Radioprotection and astrobiological studies	Pacelli et al. (2020)
*Cystobasidium* sp.	Orange	Not defined (possibly carotenoid)	Phosphate solubilization (agrochemical)	Silva et al. (2022c)
*Epicoccum* sp.	Yellow and orange	Anthraquinones and polyketide	Oxidative foliar photo-necrosis (agrochemical)	Hernández-Chacón et al. (2025)
*E. italicum (E. nigrum)*	Yellow to reddish-orange	Not defined	Production of L-ASNase (Health sector)	Andrade et al. (2023)
*Forliomyces uniseptata*	Reddish-brown	Not defined	Bioelectrochemical	Pérez-García et al. (2025)
*Fusarium* sp.	Carmine-pink	Not defined	Photosensitizing (agrochemical)	Hernández-Chacón et al. (2025)
			Mitigation of acrylamide generation - Production of L-ASNase (food sector)	Lakhdari et al. (2025)
*Monascus ruber*	Red	Not defined	Food Colorant	Darwesh et al. (2020)
*Hortaea werneckii*	Black	Melanin	Biopesticide sunscreen agent (agrochemical)	Saleh et al. (2018)
*Penicillium* sp.	Dark orange	Carotenoid (tangeraxanthin and 4 ketonostoxanthin)	Antimicrobial activity (Health sector)	Pandey et al. (2018)
	Green and yellow	Not defined	Bioelectrochemical	Silva et al. (2022a)
*Phoma* sp.	Brown–red	Melanin	Biological (antioxidant) and antimicrobial agent (agrochemical)	Surendirakumar et al. (2022)
*Pseudogymnoascus* sp.	Yellowish to orange	Amphiol	Antimicrobial activity (Health sector)	Fujita et al. (2021)
	Yellowish-white	(+)-macrosphelide a and (+)-macrosphelide b	Antibacterial and antifungal activity (Health sector)	Antipova et al. (2023)
	Yellow and pink-violaceus	Not defined	Antimicrobial, leishmanicidal and trypanocidal activity (Health sector)	Cavalcante et al. (2024)
	Not mencioned	Polyketide (bisdechlorogeodin)	Antibacterial activity against phytopathogens - citrus canker (agrochemical)	Ferrarezi et al. (2024)
*Rodosporidiobolus colostros*	Orange	Possibly carotenoid	Lignin biotransformation at low temperatures	Margesin et al. (2022)
*Sporobolomyces roseus*	Pink	Possibly carotenoid	Packaging - EPS polymeric film (Food sector)	Rusinova-Videva et al. (2024)
*Talaromyces* sp.	Orange	Not defined	Bioelectrochemical	Silva et al. (2022a)
	Reddish and yellow	Not defined	Antimicrobial activity against bacteria and yeasts (Health sector)	Lins et al. (2022)
*Talaromyces cnidii*	Pink-violaceus	Not defined	Antimicrobial activity (Health sector)	Cavalcante et al. (2024)
*Trichoderma viride*	Greenish mycelium	Anthraquinone (chrysophanol)	Antifungal activity against phytopathogens (agrochemical)	Al-Aaraji (2025)

### Agrochemical sector

Although little explored, the application of fungal pigments in agriculture is attracting interest due to its promising and sustainable potential related to bioactive properties and the protection of plant crops. This is because, with climate change, agriculture has suffered major impacts on food production associated with both abiotic and biotic factors. With temperature variations, there is greater stress that significantly affects the quality and productivity of crops on a global scale, as well as greater vulnerability to phytopathogens (Khan et al. 2020; Zhong et al. 2024). In this scenario, agrochemicals are used in large quantities for crop control and protection, leading to environmental problems and also affecting public health (Devi et al. 2022). For this reason, natural alternatives, such as fungi and their metabolites, are possible as valuable sources of biomolecules with bioactive properties that are still little explored for agricultural applications, especially when considering those taxa adapted to extreme areas (Zenteno-Alegría et al. 2024; Kim et al. 2025).

Among the classes of fungal pigments, melanins are widely known for their photoprotective potential, their ability to absorb UV radiation, and their redox/antioxidant properties, which can be used to formulate biological products based on this pigment (Muñoz-Torres et al. 2024). For example, when evaluating the potential photoprotective action of melanin produced by black yeast *Hortaea werneckii*, isolated from an Egyptian saline lake, the results indicated it as a potent radioprotective resource for *Bacillus thuringiensis* based biopesticides, protecting the formulation’s properties against the effects of UV radiation and contributing to a ninefold increase in cotton bollworm mortality (Saleh et al. 2018). Furthermore, the redox properties of microbial melanins, such as pyomelanin, produced by both bacteria and fungi, can act as mediators in the mobility and availability of iron by reducing ferric ions to their ferrous form, contributing to the synergistic action of siderophores, which are strongly related to stimulating the defensive response of plants, as well as inhibiting phytopathogens by increasing iron competitiveness in the soil (Pavan et al. 2020; Chowdappa et al. 2020; Fernandes et al. 2021; Styczynski et al. 2022).

From the perspective of soil resource bioavailability, fungal strains isolated from drylands can also serve as alternatives for formulating biofertilizers/bioinoculants, given their potential to promote plant growth and stimulate the prospecting of biomolecules for agriculture (Passarini et al. 2024; Chandra et al. 2025). An orange-pigmented yeast belonging to the genus *Cystobasidium*, isolated from an Antarctic lichen *Polycauliona regalis*, was able to solubilize phosphate in vitro, being one of the few studies to report such potential of lichen isolates from this environment (Silva et al. 2022c). Similarly, the filamentous strains *Cladosporium* sp. 1EM.P1 and *Penicillium steckii* 5Y.P4 isolated from Antarctic sediments were also able to solubilize phosphate, with larger in vitro hydrolysis halos (29.0 and 26.0 mm, respectively), and the melanized *Cladosporium* was also able to solubilize phosphorus under different temperature and salinity conditions, with maximum P levels of 106.14 mg/L (15 °C), 80.89 mg/L (25 °C) and 127.93 mg/L (0.5 M NaCl), suggesting that these isolates may act in the phosphorus cycle under different conditions in their isolation environments (Silva et al. 2025a).

Melanins can still exhibit antimicrobial activity in bacterial and fungal models, with potential applications against phytopathogens. When melanin from the endophytic fungus *Phoma* sp. RDSE17 was evaluated against human and plant pathogens, this pigment demonstrated antibacterial activity against *Bacillus subtilis*, *Staphylococcus aureus*, *Escherichia coli* and *Salmonella typhi*, and antifungal against *Aspergillus flavus*,* A. niger*, *Rosellinia* sp. and *Ustilaginoidea virens*, in addition to possessing antioxidant properties (Surendirakumar et al. 2022), which contributes to good prospects in the potential formulation of bioproducts with crop protection properties. The genus *Phoma* has also been found associated with the roots of plants in the Atacama Desert, in Chile (González-Teuber et al. 2017) and in soil and moss samples collected from Signy Island, Antarctica (Hamidu et al. 2025). From a biological control and post-harvest product preservation perspective, melanized fungi also show potential to act as biocontrol agents for phytopathogens, as demonstrated by the psychrophilic yeast *Aureobasidium subglaciale*, which was able to reduce apple fruit rot after harvest, under refrigeration, by more than 60% for *Botrytis cinerea*, and about 40% for *Colletotrichum acutatum* (Zajc et al. 2022).

Although they are more widely used in other industries (Kreusch and Duarte 2021), the class of fungal carotenoids also exhibits good active properties and can be used in the formulation of bioproducts for agricultural applications in mitigating stress factors and protecting plant tissues. They can also be used as a strategy to increase the photochemical stability of biopesticides, similar to patents that use microparticles resistant to UV radiation (Braun et al. 2019). Regarding antioxidant properties, fungal carotenoids have already been linked to the improvement of abiotic stress in plants, as they are associated with the ability to eliminate reactive oxygen species (ROS) and peroxyl (Naz et al. 2023; Basit et al. 2024). These characteristics may suggest potential applications in bioinoculants that can act as mitigators of oxidative stress and even act as a biostimulant in plant development. Furthermore, among the carotenoids, torularhodin possesses antimicrobial properties and is synthesized mainly by yeasts of the genera *Rhodotorula* and *Sporobolomyces*, being able to grow abundantly in extreme dry environments (Kot et al. 2018; Buzzini et al. 2018). Therefore, the antimicrobial activity of this type of pigment can also be evaluated against phytopathogenic microorganisms (Zajc et al. 2022).

Within the class of polyketides, anthraquinones stand out as a group of compounds with quite diverse bioactive properties, including antimicrobial and phytotoxic activities (Xu et al. 2021; Calumby et al. 2025), suggesting good prospects for agricultural use. When evaluating an anthraquinone (chrysophanol) produced by the fungus *Trichoderma viride* with greenish mycelium isolated from soils in agricultural regions of different districts of Baghdad province, this fungal metabolite was able to inhibit the growth of fungal phytopathogens such as *Fusarium oxysporum*, *F*. *solani*, *Rhizoctonia solani*, *Sclerotinia sclerotiorum* and *Pythium aphanidermatum* isolated from infected plants (tomato, eggplant, and cucumber) (Al-Aaraji 2025). Although infrequent, representatives of this genus have been detected by independent cultivation methods in the Antarctic region (Menezes et al. 2021; Passarini et al. 2024). A bisdechlorogeodin produced by an Antarctic species of the genus *Pseudogymnoascus* has shown antibacterial activity against the causative agent of citrus canker when evaluating the pigmented extract on young leaves of *Citrus sinensis*, which was able to inhibit the in vitro growth of *Xanthomonas citri* bacteria (inhibitory concentration of 90 ≈ 156 µg ml⁻¹) and reduce bacterial infection in the tested leaves from 1.55 lesions/cm² to 0.04 lesions/cm² (Ferrarezi et al. 2024).

On the other hand, aromatic polyketides such as some fungal anthraquinones and azaphilones also exhibit phytotoxic properties (Xu et al. 2021), which can be considered as sustainable alternatives to chemical herbicides in the control of unwanted plants in agricultural crops. Recently, studies have reported that some anthraquinones produced by different phytopathogenic fungi, such as some species belonging to the genera *Fusarium*, *Epicoccum*, *Alternaria*, *Phoma* and *Neopestalotiopsis*, were able to cause necrosis in plant tissues due to the ability of this type of pigment to act as photosensitizers that generate damage through oxidative necrosis, leading to tissue death (Hernández-Chacón et al. 2025, 2026). While the fungus *Chaetomium globosum*, isolated from the root of a plant (*Artemisia desterorum*) from the Tengger Desert (Ningxia Province, China), was able to produce secondary metabolites with a skeleton structurally related to spiciferone A, an azaphilone analog already reported as phytotoxic (Zhang et al. 2021).

Pigments with antioxidants, antimicrobial, nematicidal, insecticidal, or other bioactive properties reported by industries such as pharmaceuticals, cosmetics, and clinical settings may also have analogous applications in agriculture against phytopathogenic agents (Surendirakumar et al. 2022). Expanding assays that encompass the properties of fungal pigments in the agrochemical field is fundamental for providing natural resources that are less harmful to the environment, human health, agroecological relationships, and the quality of food production in the face of climate change, and fungi from more restricted areas can be a valuable source for this purpose (Zenteno-Alegría et al. 2024).

### Cosmetics sector

Fungi, as previously mentioned, adapt well to adverse conditions and synthesize secondary metabolites with antioxidant, anti-inflammatory and photoprotective properties (Cavalcante et al. 2024). These characteristics also make them promising candidates for the development of hygiene, care, and skin beautification products, aligning them with the demand for biocompatible and ecologically viable inputs (Khan et al. 2021). In this context, fungal pigments from extreme environments have demonstrated great potential for application in the cosmetic industry (Contreras-Machuca et al. 2022). In this sector, there is a particular concern about skin damage caused by frequent exposure to ultraviolet (UV) radiation, which triggers photoaging, irregular pigmentation, dryness, and burns, and is a risk factor for the development of skin cancer. Thus, the application of sunscreens is essential for preventing these damages, and the increase in their use has occurred in parallel with greater social awareness of the harmful effects of UV radiation (Oh et al. 2021b). However, chemical ingredients such as benzophenone-3, octocrylene, and cinnamate are common in sunscreens and, depending on their concentrations in the formulations, produce free radicals that damage the skin and can cause endocrine dysfunction, as well as negative environmental impacts due to their bioaccumulation in marine microorganisms and coral reef bleaching (Oh et al. 2021a; Stolecka et al. 2025). One way to avoid cellular and environmental toxicity is the use of biological pigments such as natural melanins, consisting of indolic polymers resulting from the oxidation and polymerization of phenolic compounds (Oh et al. 2021a; Pacelli et al. 2020b).

Fungi from extreme environments are excellent producers of pigments of different classes, and melanins have great potential from these eukaryotes, since they exhibit multiple properties ranging from thermotolerance and photoprotection to the elimination of free radicals and metallic ions. Due to their high UV protection and antioxidant capacity, they are used in sunscreens (Oh et al. 2021b). Furthermore, because of their ability to absorb and dissipate energy, melanins have good chemical stability (Ghadge et al. 2020), in addition to being biocompatible and exhibiting attractive coloration in cosmetics, shampoos and hair dyes (Choi 2021). Melanized fungi found in Antarctica have been the subject of research due to their excellent ability to produce different types of melanin, with resistance to gamma radiation, UV rays, and X-rays, making them strong candidates for use in the formulation of photoprotective products. These include *Friedmanniomyces endolithicus* (Coleine et al. 2020; Sajjad et al. 2020) and *Cryomyces antarcticus*, capable of surviving in space conditions and even simulations of Mars, thus serving as models for radioprotection studies of arid cryospheric lineages (Pacelli et al. 2020a), although the applications of these strains to cosmetics have not yet been significantly demonstrated.

In humans, melanin synthesis and distribution is regulated by the enzyme tyrosinase, and its disruption leads to irregular skin hyperpigmentation, so skin-lightening cosmetic products are used to mitigate this occurrence. Georgousaki et al. (2022) isolated two endophytic fungal strains (CF-090361 and CF-090766) of the genus *Comoclathris*, originating from stems of xerophytic plants (*Sedum sediforme* and *Nerium oleander*) in the Andalusian desert, Spain. From these, the authors were able to identify three new compounds with anti-tyrosinase activity, which prevents the conversion of tyrosine into melanin and thus the production of pigment in the skin. The compound identified as Comoclathrin (1) (IC50 = 0.16 µM) was 90 times more potent than the positive control kojic acid (IC50 = 14.07 µM), which is a known skin-lightening substance derived from fungi of the genera *Aspergillus* and *Penicillium*. Comoclathrin (1) also demonstrated an absence of cytotoxicity against normal (BJ fibroblasts) and cancerous cell lines, making it an excellent candidate for the development of a skin-lightening agent. Thus, based on the examples discussed, pigments produced by extremophilic fungi are promising sources for composing the formulation of functional cosmetics; the studies presented demonstrate their biological safety in association with the multifunctional properties resulting from their chemical diversity (Oh et al. 2021a). Furthermore, biotechnological techniques can allow for stability across different pH and temperature ranges, with increased resistance to photodegradation, ensuring versatility in formulations and providing safety and efficacy in prolonged use from fungal sources (Venkatachalam et al. 2023).

### Food sector

The participation of fungi and their byproducts in the food industry is recurrent and diverse, as they are commonly observed in the production of enzymes, organic acids, biofuels, and protein-rich food ingredients (Borkertas et al. 2025). For example, fermented foods and beverages are obtained through fermentation mechanisms due to the active metabolism of yeasts and molds, in processes that date back to antiquity, enabling the production of essential food items such as bread, cheese, and rice, and beverages such as beer and wine (Pouris et al. 2024). It is also worth noting that the large-scale use of fungi by the food industry results in benefits to human health. This can be seen in the production of macrofungi such as mushrooms, which are frequently obtained due to their high protein content and rich concentration of antioxidants, vitamins, dietary fiber, carbohydrates, and minerals. Due to these constituents, they exhibit antioxidant, anti-inflammatory, and antimicrobial properties. Furthermore, fungi produce bioactive compounds and enzymes with the ability to increase shelf life, nutritional value and safety, and for this reason, they are used in food processing (Pouris et al. 2024).

In this industry, fungal pigments form a fundamental segment and attract modern consumers, known for being more conscious about their diet, since synthetic pigments, although economical and less laborious to produce than natural ones, are usually less safe, due to several health problems associated with the constant use of synthetic dyes, such as allergies, sleep disorders, hyperactivity, and irritability (Poorniammal et al. 2021). In this context, fungi occupy the podium in the production of a variety of natural pigments; among the classics are β-carotene, commonly produced by the species *Blackslea trispora*, monascin, produced by the species *Monascus anka* (Elkhateeb and Daba 2023), riboflavin, produced by the species *Ashbya gossypii*, among other species (Fonseca et al. 2022). Regarding applications, these fungal pigments have extensive uses as food colorants and preservatives due to their natural properties (Lagashetti et al. 2019; Soliman et al. 2022).

In the search for new sources of these molecules, recent research shows that fungi originating from desert habitats, which are abundant in species and frequency, possess promising pigments for the food industry (Long et al. 2024). In the Antarctic region, the fungus *Geomyces* sp. WNF-15 A produces a high-quality red pigment with interesting characteristics for alternative application in the food sector when compared to pigments from *Monascus* and cochineal, including excellent water solubility, color stability across a wide pH range, tolerance to ultraviolet rays, oxidants, and metallic ions, in addition to being non-toxic (Huang et al. 2020; Long et al. 2024). From hot desert areas, such as the Eastern Province of Saudi Arabia, two reddish fungal species in cultivation, *Achaetomium cristalliferum* and *Chaetomium strumarium*, have had their pigmented extracts show potential in providing heat stability and color retention when added to white rice and puff pastry after cooking (Alyami et al. 2025). In the Egyptian region, also known as *Monascus ruber* (OMNRC45), a red-colored fungus produced a pigment considered biosafe, without the production of citrinin in its fermentation process and non-toxic in mouse models (*Mus musculus domesticus*) compared to the synthetic carmoisine, and applicable as a food coloring in the manufacture of lollipops and jellybeans. In addition to the observed safety, sensory characteristics such as color, appearance, and overall acceptability when incorporated into these products were considered improved by the use of this natural fungal dye (Darwesh et al. 2020).

Due to their hepatotoxic and nephrotoxic effects, the removal of mycotoxins during fermentation processes is essential for the use of fungal pigments in foods. Red-mold rice (RMR) fermented by different species of the genus *Monascus* spp., for example, is considered a lipid-lowering functional food due to monacolin K, an inhibitor of de novo cholesterol synthesis. However, the presence of citrinin poses a challenge in the fermentation process, and techniques such as phosphate-ethanol extraction (45% ethanol, 1.5% phosphate, and 70 min of extraction) have shown good results for citrinin removal, achieving a 91.6% removal rate (Lee et al. 2007). Various alternatives for controlling the production medium have been refined to mitigate the production of this mycotoxin by *Monascus* spp. (Farawahida et al. 2022). Furthermore, a positive relationship was identified between NaCl supplementation in the cultivation of *Monascus* purpureus—a fungus widely used in Asian cuisine—and a subsequent reduction in citrinin content, with no noticeable change in fungal growth but a notable increase in pigment production (Zhen et al. 2019).

In current safer food models, another possibility for the use of extremophilic fungal strains in the food sector is the reduction and/or mitigation of acrylamide generation, a toxic compound with carcinogenic and genotoxic risk produced after heat induction during the thermal processing of foods rich in sugars. One strategy for this has been the screening of stable L-asparaginase-producing fungal strains (Ashok et al. 2019; Thakur et al. 2025). For example, Lakhdari et al. (2025) reported the endophytic strain of *Fusarium* sp3, found in the leaves of *Malcolmia aegyptiaca* Spreng. in southeastern Algeria, capable of producing this enzyme (63.68 U/mL) that hydrolyzes and reduces levels of asparagine, one of the precursor compounds in the formation of acrylamide, before thermal processing in food, thus having the potential for efficient use in preventing its formation, and therefore having high potential for this application.

The Antarctic rose yeast *Sporobolomyces roseus* AL103, originating from Livingston Island, has shown promising characteristics for an innovative application in the food industry, producing exopolysaccharides (EPS) that can be used in the manufacture of a polymeric film for food packaging (Rusinova-Videva et al. 2024). Characteristics such as gas and water vapor permeability, color, and mechanical properties were tested and determined according to its thickness, as these aspects can determine the shelf life of the packaged food. The results demonstrated that EPS from this fungus can be efficiently used in packaging manufacturing, as it exhibits satisfactory mechanical, thermogravimetric, and moisture retention performance. Furthermore, it was shown that, with some optimization adjustments in EPS production, it is possible to obtain a product with greater economic viability (Rusinova-Videva et al. 2024).

Many challenges to the applicability of promising extremophilic fungal strains in the food industry are noteworthy, whether due to characteristics related to their ideal metabolic functioning temperature, as in the case of psychrotolerant strains that only produce the promising pigment within an extremely restricted range of low temperatures, and/or due to the release of mycotoxins that can have negative effects such as hepato- and nephrotoxicity. These limitations restrict application and require strategies to overcome these obstacles (Poorniammal et al. 2021). It is evident, therefore, that the use of fungi in the food industry is promising, presenting itself as an alternative to reduce the consumption of synthetic products that are often considered harmful to human health. Pigmented fungi from extremophilic environments deserve attention, given their wide range of applicability in the food industry, whether as colorants, by reducing the production of undesirable compounds, or by producing packaging, as seen here.

### Pharmaceutical and/or health sector

Fungi are excellent producers of bioactive metabolites with medicinal properties such as antioxidant, antibacterial, antiparasitic, antifungal, anticancer, and other biological activities already reported in previous studies (Wang et al. 2022). Among fungal metabolites, pigments from extremophilic fungi have great potential for health applications, as they combine their bioactive activities with lower toxicity when compared to synthetic substances (Toma et al. 2023). Cryospheric environments have been the target of searches for fungi that produce pigments with medicinal activity, a fungal strain of *Penicillium* sp., from soils in the Indian Himalayas, a producer of orange pigment containing different carotenoid derivatives that demonstrated antimicrobial activity against actinobacteria (*Streptomyces* sp., *Nocardia tenerifensis*), Gram-positive bacteria (*Bacillus subtilis*, *B*. *megaterium*) and Gram-negative bacteria (*E. coli*, *Pseudomonas putida*, *Serratia marcescens*) (Pandey et al. 2018).

Predominant in polar regions of the globe, such as the Arctic and Antarctic, the fungal genus *Pseudogymnoascus*, isolated from a soil sample collected in Tokyo, Japan, stands out for producing significant pigments (Villanueva et al. 2021; Satriawan et al. 2024), such as amphiol, yellowish to orange in color, with antifungal activity against *Candida albicans* and *Aspergillus fumigatus* (strain PF1464) (Fujita et al. 2021), and *pseudogymnoascins*, yellow in color, with antibacterial activity against *S. aureus*, *B. subtilis*, *E. coli* and *P. aeruginosa*, and antifungal activity (Antipova et al. 2023). Also with strains of this genus, Cavalcante et al. (2024) reported *Pseudogymnoascus* sp. with relevant antimicrobial activities. Two strains inhibited *S. aureus*, with the yellow-pigmented strain SC04.P3 and the pink/violet strain ACF093, while two other pink/violet strains (SC12.P3 and SC32.P3) showed leishmanicidal activity against *Leishmania infantum* and *L. amazonenses*, and trypanocidal activity against *Trypanosoma cruzi*. The pigmented extract of *Talaromyces* sp. isolates from arid areas, ranging in color from red, yellow, and pink-violet, showed interesting antimicrobial activity against bacterial and yeast-like targets (Lins et al. 2022; Cavalcante et al. 2024).

Antitumor effects of fungi have been reported, for example, in research seeking alternative L-asparaginase for chemotherapeutic use in acute lymphoblastic leukemia (ALL), an aggressive neoplasm affecting the bone marrow, and common in children (Golbabaie et al. 2020). Researchers have found fungi that produce this enzyme, among them pigmented fungi such as *Epicoccum italicum*, yellow to reddish-orange, isolated from Antarctica, which produces the enzyme free of glutaminase and urease side effects, potentially being more selective and less cytotoxic (Andrade et al. 2023). Other Antarctic fungi have also been reported as important enzyme producers of this type (Lima et al. 2022), for example, the creamed yeasts *Leucosporidium muscorum* and *L*. *scottii* L115 exhibited L-asparaginase activity associated with lower glutaminase activity (Freire et al. 2020; Sánchez-Moguel et al. 2023), exemplifying the power of extremophilic fungi in the medical field.

### Textile sector

The textile industry is one of the most significant sectors in the global economic market. Increased population demand and the discovery of synthetic dyes have driven the large-scale production of textile materials (Dutta et al. 2022; Yadav et al. 2023). Structurally, the transformation of raw materials such as wool, cotton, silk, and polyester into the final product follows the stages of yarn formation, fabric construction, wet processing, the dyeing stage, and the final product, which vary according to the different purposes (Khan 2025). However, wet processing is the stage with the greatest environmental impact due to the disposal of textile effluents and high water consumption (Dutta et al. 2022). Although synthetic dyes have commercial advantages such as low cost, durability, and yield, pigments of synthetic origin can be highly toxic, contain carcinogenic chemicals, and are not biodegradable, which poses a risk to biodiversity (Yadav et al. 2023).

A sustainable alternative to reduce environmental risks and health impacts is the use of microbial-derived colorants. Besides being an ecological option, many microorganisms offer properties of biotechnological interest, easy laboratory cultivation, chemical stability during industrial processes, and a blend of bioactive properties. These attributes mark exceptional activities for the biotechnology industry, such as the production of antioxidants, the creation of photoprotective products, antimicrobial and antifungal actions for the pharmaceutical sector, applications in cosmetics and the food industry. In the textile sector, the interest lies in the aforementioned attributes and the high coloring power with affinity for natural fibers, which increases productive efficiency (Chadni et al. 2017; Sajjad et al. 2020; Basto et al. 2025; Taware 2025). Pigments from fungi such as *Monascus*, *Penicillium*, *Aspergillus*, *Fusarium*, *Talaromyces*, *Trichoderma*, *Neurospora*, *Alternaria* and *Cordyceps* are promising for dyeing fibers such as cotton, wool, and silk (Lin and Xu 2022; Cavalcante et al. 2024; Elkhateeb and Daba 2023; Taware 2025).

Although mesophilic fungi already show great potential, the landmark of biotechnological innovation is present for arid extremophilic strains (Sepe et al. 2025). These microorganisms have been extensively investigated due to their metabolic activities and adaptations to extreme environmental conditions, such as pH levels, salinity, temperature, and nutrient scarcity (Cavalcante et al. 2024), exhibiting interesting properties for the field of textiles. For example, filamentous fungi identified as *Achaetomium cristalliferum* and *Chaetomium strumarium*, presented intense pigments in red and reddish-brown, respectively (Alyami et al. 2025). The samples exhibited high thermal stability in their extracellular pigments, which maintains the coloration of the fabric after industrial processes and numerous washes. Tests to determine the toxicity of the extracts were satisfactory, indicating minimal toxicity at high concentrations, excellent for biotechnological application. With an emphasis on the textile industry, wool was the raw material chosen for dyeing tests, which yielded remarkable results and could be very promising given its high fixation capacity even after many processes that would influence the pigment’s hue. Another advantageous aspect of these studied fungi is the efficiency in pigment extraction, which proved to be more productive using water as a solvent, indicating the water-soluble nature of the compounds produced, reducing costs and being environmentally beneficial, ideal for large-scale production, in addition to exhibiting antibacterial and antifungal capacity (Alyami et al. 2025). These properties are noteworthy and add commercial value, suggesting their use in suture threads, face masks, hospital clothing, sportswear and underwear (Samanta 2020; Elkhateeb and Daba 2023).

On the other hand, studies of the potential of cryospheric fungal pigments for the manufacture of fabrics with photoprotective and antioxidant properties have also been observed. Simonetti et al. (2024) reported dark brown to black melanized fungal species of the genus *Cladosporium*, isolated on King George Island in Antarctica, with high resistance to UVB and UVC radiation and capable of surviving in high salinity levels with up to 15% NaCl supplemented in the culture medium. These characteristics may be linked to microorganisms adapted to the environment, in addition to the presence of melanized pigments due to their photoprotective mechanism that helps these producing fungi survive in environments with high radiation levels such as Antarctica (Sajjad et al. 2020). Similarly, the study by Pandey et al. (2018) analyzed the fungus *Penicillium* sp. (GBPI_P155), the extracellular pigment is orange in color and of the carotenoid type, a secondary metabolite resulting from environmental stress. The fungus in question produced pigment at temperatures between 15 °C and 35 °C, with the highest yield at 15 °C, in addition to excellent tolerance to pH levels ranging from more acidic (pH 2) to more alkaline (pH 14). Consequently, its stability under high physicochemical changes is attractive to textile dyeing factories. During the dyeing stages, much dye is lost due to these variations, which affect the color intensity, leading to the addition of more pigment. Many of these pigments are highly toxic and non-biodegradable, causing water pollution. Maintaining the dye’s properties even after many industrial processes indicates less excessive waste, as well as a more sustainable option (Basto et al. 2025).

### Other industries and possible uses

Pigmented fungi from extreme cold and hot areas and their cellular metabolisms also find potential in alternative uses. In the environmental field, they are important for bioremediation strategies or as bioindicators, thus having interesting ecological and biotechnological significance (Margesin et al. 2022). For example, the reddish yeast *Rhodosporidiobolus colostri*, from alpine forest soil, showed potential in the biodegradation of lignin-derived compounds, such as p-coumaric, ferulic, and 4-hydroxybenzoic acids, using them as a carbon source even under low temperature conditions (1–25 °C). Additionally, the study evidenced a co-substrate-dependent metabolic behavior (such as vanillic acid), which were metabolized only in the presence of other substrates, while others, such as syringic acid, were not metabolized, indicating chain reactions that are the result of complex metabolic interactions and incredible metabolic specificity (Margesin et al. 2022). This demonstrates that pigmented psychrotrophic yeasts can act metabolically as secondary agents in the degradation of compounds derived from lignin, playing a central role in the assimilation and detoxification of aromatic compounds, which broadens the interest for both extreme ecosystems and the development of sustainable biotechnology.

Furthermore, pigments from extreme fungi may be promising in the field of bioelectrochemistry and electricity generation (Silva et al. 2022a; Pérez-García et al. 2025). The pigment from the fungus *Forliomyces uniseptata* – a fungus that produces a reddish to reddish-brown pigment, a characteristic associated with the presence of chromophores with high spectral absorption capacity – has been reported for this potential, exhibiting relevant electrochemical properties characterized by a reversible redox cycle, which allows its use as a redox mediator in microbial fuel cells (MFCs) (Pérez-García et al. 2025). The reversible redox behavior was evidenced by cyclic voltammetry analyses, which indicated its ability to alternate between oxidized and reduced states in a stable manner. In this system, the pigment acted as a natural electronic mediator, receiving electrons from microbial metabolism and subsequently transferring them to the electrode, allowing for the continuous generation of electric current. The electrochemical curves showed the reversibility of the reaction, demonstrating its efficiency in extracellular electron transfer with performance similar to that of synthetic meters that are traditionally used, reinforcing the potential of these fungal metabolites as viable and sustainable alternatives for generating electricity and bioelectrochemical systems (Pérez-García et al. 2025). The pigment from this fungus was applied in a system inoculated with *Bacillus subtilis*, resulting in the generation of bioelectricity with a maximum power density of approximately 37 µW/cm². Observing the environmental factors, it was noted that the incidence of light directly influenced the biosynthesis of the pigment, with blue light having the ability to accelerate its production in this system (Pérez-García et al. 2025).

In a complementary way, the study by Silva et al. (2022a) demonstrated that some fungal pigments can play an important role in bioelectrodes, not only by generating electricity but also by stimulating the activity of oxidase enzymes associated with electrochemical processes. It was observed that the fermentative addition of natural pigments (10 ppm) from filamentous fungi of the semi-arid Brazilian Caatinga, *Aspergillus* sp. (red), *Penicillium* sp. (green, yellow) and *Talaromyces* sp. (orange), contributed to the induction of extracellular production of oxidative enzymes (laccase, tyrosinase, or peroxidase, involved in the oxidation of pyrogallol to purpurogalin) by the fungi *Rhizopus microsporus var. chinensis* (UCP 1296) and *Penicillium* sp. UCP1286, when compared to the effect of the synthetic dyes methylene blue and bromothymol blue, which act in electron transfer. Through this activity, the possibility of fungal pigments as facilitators of electron transfer processes in bioelectrochemical systems is noted, reinforcing the alternative potential of these natural secondary metabolites for application in biofuel cells and technologies for sustainable energy generation (Silva et al. 2022a).

Another application of pigmented fungi from extreme arid areas is in radioprotection and astrobiological studies. The black extremophile fungus is characteristic of the restrictive Antarctic environment, *Cryomyces antarcticus*, was investigated for its resistance in Martian regolith simulants, where it was exposed to high doses of accelerated helium ion radiation, one of the main components of cosmic radiation incident on the Martian surface (Pacelli et al. 2020a). This microorganism demonstrated high tolerance to ionizing radiation, with significant maintenance of cell viability even after exposure to extreme doses. This behavior is directly associated with the presence of the compound melanin in the cell wall of this fungus, which exerts a radioprotective function through the absorption and dissipation of ionizing energy and is also related to the neutralization of reactive oxygen species generated by radiation, favoring the survival of microorganisms in these extreme space environments (Pacelli et al. 2020a). Thus, melanized fungi are relevant models in astrobiology, not only for their ability to resist radiation, but also for the potential application of the melanized pigment in radioprotection and bioremediation strategies, including the adsorption of metals and radionuclides, as well as the degradation of toxic organic compounds, such as petroleum (Cordero et al. 2017). These findings broaden the perspectives for the use of these extremophilic organisms and their metabolites for the development of technologies aimed at space exploration and the mitigation of highly irradiated environments.

## Conclusions and perspectives

Whether due to low or high temperatures, arid regions are major hotspots of fungal biodiversity under extreme conditions yet remain underrepresented and represent crucial areas for advancing the biological study of their resident strains. Based on the literature records consulted here, fungi derived from these regions, particularly microfungi, originating from substrates such as soils, plant parts, rocks, ice, snow, and glaciers, are morphologically diverse, with vibrant colors and endocellular and diffusible pigmentation, corresponding to chromatic molecules that absorb distinct ranges of visible light. In both hot and cryospheric arid environments, filamentous ascomycete taxa are frequently recovered in microbial isolations, showing colonies with dark-brown-gray, yellowish-green, orange, and bluish combinations, with extracellular pigments mainly within the spectral range of yellows, cinnamon and reddish pink. At the other extreme, orange and pink basidiomycete yeasts were more frequently recorded. The prevalence of representatives of colorful cultivable microfungi affiliated with Ascomycota and Basidiomycota is expected, due to the greater knowledge and diversity known for both, with many intrinsic biological strategies for environmental multitolerance (Santiago et al. 2018; Rosa et al. 2019), with many of the ascomycetes being more favorable in the high production of varied pigments (Elkhateeb and Daba 2023).

From a taxonomic point of view, it is valid to understand the main genera and species of these areas, and it is essential to assemble collections of mycological cultures with pigmented fungi from hot and cold dry areas, and/or to explore groups already preserved in known collections for this purpose. The formalization of an online database with these and other colored fungi also facilitates the process, as has been suggested in other documents (Elkhateeb and Daba 2023). However, even exhibiting varied colorimetric patterns, the investigation of the chemical nature of compounds in extremophilic fungi has not yet been carried out in depth in most taxa, much less the elucidation of biosynthetic pathways, since many studies are still in their early research stages within the screening process, and even when recovering different pigmented and bioactive fungi, the chemical structure and type of pigment are in many cases still not fully clarified. On the other hand, among the studies that have advanced these investigations by providing downstream steps, the pigmented molecules that confer their colors belong to the categories of melanins and carotenoids, along with azaphilones. The colorful characteristics of these fungi are linked to the production of a mixture of biomolecules (including pigment pathways) that are intimately related to the movement of environmental adaptation to the contexts of extreme arid regions, which offer a multifactorial condition of harsh temperatures, limited liquid water, abundant solar radiation, and low humidity levels. This is also evidenced among genome investigations that have revealed mainly classic pathways for the production of melanized pigments among fungi from these areas, in addition to evidence of other adaptations besides pigmentation.

Beyond their adaptive ecological and taxonomic value, pigmented strains from extreme arid ecosystems and their biosynthetic capabilities are remarkable for their potential applications, offering numerous possibilities in food, pharmaceutical, agrochemical, cosmetic, textile, optoelectronic, environmental, and even astrobiological fields, as discussed here. This is due to a wide variety of potent natural properties such as good stability (light, thermal), color fixation, non-toxicity, and protective effects against light damage, oxidizing free radicals, and pathogenic microorganisms. Many of these functions require further understanding and exploration among the different systematic groups of extreme fungi, representing a challenging path for their effective use in the sectors being bioprospected, especially to offer advantages over industrially available options from other sources. It is expected that the bioprospective field with these microeukaryotes will gain more notoriety in applied science.

In this sense, there are many ramifications to this prospecting, and the standardization of a global regulatory process, as well as the patent and commercial procedures for the use of its bioactive metabolites, are the main areas that require development and progress for this purpose (Cavalcante et al. 2023). Other challenges are also present, ranging from the recovery of colored strains from the most extreme and still unknown dry areas of the planet, to the obtaining and isolation of promising biometabolites from active cultures that are free of cellular mycotoxins co-produced in the process, therefore with the biotechnological seal of GRAS (Generally Recognized As Safe), have satisfactory yields that facilitate scaling up, and are adaptable to various conditions, including activity at higher temperatures, in the case of psychrotolerant colored fungi from cold habitats. Combining methodological isolation initiatives, taxonomic data, genome exploration and the use of robust omics techniques (use of mutagenesis, CRISPR/Cas9, RNAi) could contribute to the bioprospective plan for extremophilic fungi and their products, expanding research into elucidating all the hidden potential they possess. From this perspective, it is significant to highlight the fundamental potential that screening pigmented strains from extremely dry biomes has for generating knowledge and more sustainable applications, derived from the functional and chemical diversity of their biometabolites, especially considering a planet in transformation and a bioeconomy based on new, more socially acceptable natural solutions.

## Supplementary Information

Below is the link to the electronic supplementary material.


Supplementary Material 1


## Data Availability

All data supporting the findings of this study are available within the paper and its Supplementary material.
