# Supplementary material for: Pigmented fungi derived from hot and cold deserts ecosystems: taxonomy, genomic and biotechnological approach
Source: World J Microbiol Biotechnol. 2026 Jul 31;42(8):437. doi: 10.1007/s11274-026-05160-0 (PMC13427916; doi:10.1007/s11274-026-05160-0)
Supplement: Supplementary file 1 — Supplementary Material 1 [file 11274_2026_5160_MOESM1_ESM.docx]

**Supplementary Table 1** - Taxonomic profile of the main pigmented fungi from extreme cold and hot arid ecosystems.

| **Fungi (Basionym and current nomenclature according to NCBI taxonomy)** | **Geographic origin** | **Environmental substrate** | **Color Pigment** | **Type Pigment** | **Chemical category** | **Bioactivity** | **References** |
| --- | --- | --- | --- | --- | --- | --- | --- |
| **Hot arid ecossystem** | | | | | | | |
| **Ascomycota** | | | | | | | |
| *Achaetomium cristalliferum* | Eastern Province of Saudi Arabia | Soil and air | Red | Extracellular | Not described | Dyeing; antibacterial and antifungal effect | Alyami et al. (2025) |
| *Alternaria alternata* | Jordanian Desert | Soils and parts of fruit | Dark brownish | Possibly intracellular (but not formally described) | Melanin | Not described | Alsohaili and Bani-Hasan (2018) |
|  | Saudi Arabian Desert | Soils | Dark brownish | Possibly intracellular (but not formally described) | Melanin | Not described | Ameen et al. (2022) |
| ***Alternaria chlamydosporigena*** | Saudi Arabian Desert | Soils | Dark brownish | Intracellular | Melanin | Antioxidant effect (Flavonoids) | Ameen et al. (2022) |
| *Alternaria gaisen* | Jordanian Desert | Soils and parts of fruit | Dark brownish | Intracellular | Melanin | Not described | Alsohaili and Bani-Hasan (2018) |
| *Alternaria tenuissima* (***Helminthosporium tenuissimum*)** | Jordanian Desert | Soils and parts of fruit | Dark brownish | Intracellular | Melanin | Not described | Alsohaili and Bani-Hasan (2018) |
| *Aspergillus* sp. | Brazilian semi-arid Caatinga | Soil | Red | Not described | Not described | Bioelectrochemistry | [Silva et al. (2022)](https://doi.org/10.33448/rsd-v11i6.28799) |
| *Aspergillus niger* | Saudi Arabian Desert | Soils | Dark brownish | Intracellular | Melanin | Antioxidant and enzymatic effects | Ameen et al. (2022) |
|  | Jordanian Desert | Soils and parts of fruit | Dark | Intracellular | Possibly melanin | not described | Alsohaili and Bani-Hasan (2018) |
| *Aspergillus oryzae* | Saudi Arabian Desert | Soils | Greenish-yellowish | Intracellular | Not described | Antioxidant and enzymatic effects | Ameen et al. (2022) |
| *Aspergillus phoenicis* | Saudi Arabian Desert | Soils | Dark-brownish | Intracellular | Melanin | Antioxidant and enzymatic effects | Ameen et al. (2022) |
| *Aspergillus terreus* | Eastern Province of Saudi Arabia | Soil and air | Yellow | Extracellular | Not described | Not described | Alyami et al. (2025) |
|  | Saudi Arabian Desert | Soils | Greenish-yellowish | Not described | Not described | Antioxidant and enzymatic effects | Ameen et al. (2022) |
| *Aureobasidium melanogenum* XJ5-1 | Taklimakan Desert | Soil | Brown | Intracellular | Melanin | Tolerance to multiple stressors (heat, acidity and desiccation, UV radiation, high oxidation of H₂O₂ and high salt concentrations) | Jiang et al. (2016) |
| *Chaetomium madrasense* | Saudi Arabian Desert | Soils | Dark-brownish | Intracellular | Melanin | Antioxidant and enzymatic effects | Ameen et al. (2022) |
| *Chaetomium strumarium* | Eastern Province of Saudi Arabia | Soil and air | Red | Extracellular | Not described | Dyeing; antibacterial and antifungal effect | Alyami et al. (2025) |
| *Cladophialophora carrionci (Cladosporium carrionii)* | Saudi Arabian Desert | Soils | Dark | Intracellular | Possibly melanin | Antioxidant and enzymatic effects | Ameen et al. (2022) |
| *Curvularia hawaciensis (C. hawaiiensis)* | Saudi Arabian Desert | Soils | Black | Intracellular | Melanin | Antioxidant and enzymatic effects | Ameen et al. (2022) |
| *Curvularia nicotiae* | Saudi Arabian Desert | Soils | Black | Intracellular | Melanin | Antioxidant and enzymatic effects | Ameen et al. (2022) |
| *Exophiala* sp. | Atacama Desert | Soil from high-altitude volcanic area | Dark brown | Intracellular | Melanin | Resistant to UV radiation | [Pulschen et al. (2015)](https://doi.org/10.1002/mbo3.262) |
| *Fusarium brachygibbosum* | Saudi Arabian Desert | Soils | Purplish-pink | Intracellular | Not described | Antioxidant and enzymatic effects | Ameen et al. (2022) |
| *Fusarium oxysporum* | Jordanian Desert | Soils and parts of fruit | Purplish | Intracellular | Not described | Not described | Alsohaili and Bani-Hasan (2018) |
|  | Saudi Arabian Desert | Soils | Purplish-pink | Intracellular | Not described | Enzymatic effects | Ameen et al. (2022) |
| *Fusarium solani* | Eastern Province of Saudi Arabia | Soil and air | Yellow | Extracellular | Not described | Not described | Alyami et al. (2025) |
| *Neocatenulostroma* sp. | Atacama Desert | Soil | Dark brown | Intracellular | Melanin | Not described | Culka et al. (2017) |
| *Penicillium* sp. | Brazilian semi-arid Caatinga | Soil | Green and yellow | Not defined | Not defined | Bioelectrochemical | [Silva et al. (2022)](https://doi.org/10.33448/rsd-v11i6.28799) |
| *Penicillium citrinum* | Jordanian Desert | Soils and parts of fruit | Greenish | Intracellular | Not described | Not described | Alsohaili and Bani-Hasan (2018) |
| *Penicillium chrysogenum* | Saudi Arabian Desert | Soils | Greenish | Intracellular | Not described | Antioxidant and enzymatic effects | Ameen et al. (2022) |
| *Penicillium pinophilum* (***Talaromyces pinophilus*)** | Coahuila semi-desert, Mexico | Leaves (*Quercus* spp. and *Larrea tridentata*) | Red-purple | Extracellular | Not described | Not described | [Espinoza-Hernández et al. (2013)](https://doi.org/10.4314/ajb.v12i22.) |
| *Penicillium purpurogenum* (***Talaromyces purpureogenus*)** | Coahuila semi-desert, Mexico | Leaves (*Quercus* spp. and *Larrea tridentata*) | Orange-red | Extracellular | Not described | Not described | [Espinoza-Hernández et al. (2013)](https://doi.org/10.4314/ajb.v12i22.) |
| *Stemphylium solani* | Saudi Arabian Desert | Soils | Dark-brownish | Intracellular | Possibly melanin | Antioxidant and enzymatic effects | Ameen et al. (2022) |
| Talaromyces sp. | Brazilian semi-arid Caatinga | Soil | Reddish and yellow | Extracellular | Not described | Antimicrobial activity against bacteria and yeasts. | [Lins et al. (2022)](https://doi.org/10.33448/rsd-v11i11.33045) |
|  |  | Soil | Orange | Not described | Not described | Bioelectrochemistry | [Silva et al. (2022)](https://doi.org/10.33448/rsd-v11i6.28799) |
| *Talaromyces variabilis* | Saudi Arabian Desert | Soils | Greenish | Intracellular | Not described | Antioxidant and enzymatic effects | Ameen et al. (2022) |
| *Trichoderma longibrachiatum* | Saudi Arabian Desert | Soils | Greenish | Intracellular | Not described | Antioxidant and enzymatic effects | Ameen et al. (2022) |
| *Ulocladium* sp. (*Alternaria* sp.) | Saudi Arabian Desert | Soils | Brown, olive green | Intracellular | Not described | Enzymatic effects | Ameen et al. (2022) |
| **Basidiomycota** | | | | | | | |
| *Cryptococcus friedmannii (Naganishia friedmannii)* | Atacama Desert | Soil from high-altitude volcanic area | Pale-beige | Intracellular | Carotenoid | Resistant to UV radiation | [Pulschen et al. (2015)](https://doi.org/10.1002/mbo3.262) |
| *Holtermanniella wattica (Cryptococcus watticus)* | Atacama Desert | Soil from high-altitude volcanic area | Pale-beige | Intracellular | Carotenoid | Resistant to UV radiation | [Pulschen et al. (2015)](https://doi.org/10.1002/mbo3.262) |
| *Rhodotorula colostri* (*Rhodosporidiobolus colostri*) | Baghdad, Iraq - Middle East | Soil | Orange | Intracellular | Possibly Carotenoid | Not described | [Al-Atrash et al. (2021)](https://doi.org/10.18502/ijm.v13i3.6406) |
| *Rhodotorula laryngis (Cystobasidium laryngis)* | Baghdad, Iraq - Middle East | Soil | Orange | Intracellular | Possibly Carotenoid | Not described | [Al-Atrash et al. (2021)](https://doi.org/10.18502/ijm.v13i3.6406) |
| *Rhodosporidium babjevae (Rhodotorula babjevae)* | Baghdad, Iraq - Middle East | Soil | Orange | Intracellular | Possibly Carotenoid | Not described | [Al-Atrash et al. (2021)](https://doi.org/10.18502/ijm.v13i3.6406) |
| *Rhodosporidium toruloides (Rhodotorula toruloides)* | Atacama Desert | Soil from high-altitude volcanic area | Orange | Intracellular | Carotenoid | Resistant to UV radiation | [Pulschen et al. (2015)](https://doi.org/10.1002/mbo3.262) |
| **Mucoromycota** | | | | | | | |
| Rhizopus stolonifer | Jordanian Desert | Soils and parts of fruit | Dark and brownish | Intracellular | Possibly melanin | Not described | Alsohaili and Bani-Hasan (2018) |
| **Cold arid ecossystem** | | | | | | | |
| **Ascomycota** | | | | | | | |
| Alternaria sp. | Antarctica | Mosses (endophytes) | Black-brown | Intracellular | Possibly melanin | Enzyme production (L-ASNase) | Andrade et al. (2023) |
| Aspergillus sp. | Antarctica | Mosses (endophytes) | Greenish; brown | Intra and extracellular | Not described | Enzyme production (L-ASNase) | Andrade et al. (2023) |
| *Antarctomyces pellizariae* | Antarctica | Snow | Blue | Intracellular | Not described | Not described | de Menezes et al. (2017) |
| *Cladosporium* sp. | Antarctica | Mosses (endophytes) | Black-brown | Intracellular | Possibly melanin | Enzyme production (L-ASNase) | Andrade et al. (2023) |
| *Cryomyces antarcticus* (CCFEE 534, CCFEE 515, MNA-CCFEE 515) | Antarctica | Rocks | Black-brown | Intracellular | Melanin (DHN, L-DOPA) | Protection against UV and ionizing radiation, tolerance to extreme environmental stresses (drought, salinity, and extreme temperatures). | Selbmann et al. (2005), Pacelli et al. (2020), Catanzaro et al. (2024) |
| *Diaporthe* sp. | Antarctica | Mosses (endophytes) | Creamy-grayish-brown | Intracellular | Not described | Enzyme production (L-ASNase) | Andrade et al. (2023) |
| *Epicoccum* sp. | Antarctica | Mosses (endophytes) | Yellow to reddish-orange | Intra and extracellular | Not described | Enzyme production (L-ASNase) | Andrade et al. (2023) |
| *Fusarium* sp. | Antarctica | Mosses (endophytes) | Reddish-orange | Intracellular | Not described | Enzyme production (L-ASNase) | Andrade et al. (2023) |
| *Geomyces* sp. WNF-15A | Antarctica | Not described | Red | Extracellular | Not described | The study addresses genetic synthesis and adaptation to normal temperature for efficient red pigment (AGRP) production, targeting food/cosmetic uses. | Long et al. (2024) |
| *Knufia obscura* | Antarctica | Soil | Black-brown | Intracellular | melanin | Not described | Isola et al. (2022) |
| *Knufia victoriae* | Antarctica | Soil | Black-brown | Intracellular | melanin | Not described | Isola et al. (2022) |
| *Penicillium* sp. (GBPI_P155) | Indian Himalayan region | High altitude soil | Orange | Intracellular (possibly, yet not described) | Carotenoids | Antimicrobial activity | Pandey et al. (2018) |
| *Penicillium* spp. | Drass Valley, Indian Himalaya | Soil | Varying between green-bluish, blue-green, and grayish tones | Not described | Not described | Not described | Nonzom and Sumbali (2021) |
| *Pseudogymnoascus* sp. (AKSP3), (AKSP4), (HNDR4), (HNDR2) | King George Island (Antarctica), Hornsund (Svalbard) | Soil | Reddish-pink | Intracellular | Not described | Pigments tested for biotechnological potential as natural dyes, potential biological activities (antioxidant/sunscreen). | Wong et al. (2022) |
| *Pseudogymnoascus* spp. (SC04.P3, SC12.P3, SC32.P3, SC122.P3) | Collins Glacier (Antarctica) | Soil | Various shades (including pink/violet, possibly others) | Extracellular | Not described | Inhibited *S. aureus*; SC12.P3 and SC32.P3 also active against *Leishmania* and *T. cruzi*. | Cavalcante et al. (2024) |
| *Pseudogymnoascus antarcticus* | Antarctica | Sponge | Yellow | Extracellular | Not described | Not described | Villanueva et al. (2021) |
| *Pseudogymnoascus lanuginosus* | Antarctica | Sponge | Cinnamon | Extracellular | Not described | Not described | Villanueva et al. (2021) |
| *Pseudogymnoascus verrucons* | Antarctica | Sponge | Purplish red | Extracellular | Azaphilone | Protection against UV/sun radiation and oxidative stress, and aid in maintaining membrane fluidity in cold conditions. | Palma et al. (2024) |
| *Talaromyces cnidii* SC34.P3 | Collins Glacier (Antarctica) | Soil | Red/orange | Extracellular | Not described | Antimicrobial activity against *Staphylococcus aureus* | Cavalcante et al. (2024) |
| *Taphrina antarctica* | Antarctica | Rock (endolithic) | Pale pink | Intracellular | Not described | Not described | Selbmann et al. 2014 |
| *Thelebolus microsporus* | Antartica | Soil | Bright orange to yellow-orange | Intracellular | Carotenoid (β-carotene) | Not described | Singh et al. (2014) |
| *Victoriomyces antarcticus* | Antartica | Soil | Red | Extracellular | Not described | Not described | Davolos et al. (2019) |
| **Basidiomycota** | | | | | | | |
| *Camptobasidium arcticum* | Greenland and Svalbard | Glacial snow | Pinkish | Intracellular | Not described | Not described | Perini et al. (2021) |
| *Dioszegia* sp. | Alps, Italy | Alpine glacier | Pinkish to orange | Intracellular | Carotenoids | Not described | Amaretti et al. (2014) |
| *Psychromyces glacialis* | Greenland and Svalbard | Glacial snow | Pinkish | Intracellular | Not described | Not described | Perini et al. (2021) |
| *Rhodotorula frigidialcoholis* | Antarctica | Permafrost | Orange, yellowish | Intracellular | Carotenoids (torularodin and β-carotene) | Not described | Touchette et al. (2021) |
| *Rhodotorula laryngis (Cystobasidium laryngis)* | Alps, Italy | Alpine glacier | Pinkish to orange | Intracellular | Carotenoids | Not described | Amaretti et al. (2014) |
| *Rhodotorula mucilaginosa* | Alps, Italy | Alpine glacier | Pinkish to orange | Intracellular | Carotenoids | Not described | Amaretti et al. (2014) |
| *Rhodotorula svalbardensis (Rhodotorula* sp. *'svalbardensis')* | Arctic | Cryoconite holes | Pale pink (mature colony) | Intracellular | Carotenoids | Production of cold enzymes | Singh et al. (2014) |
| *Rhodosporidiobolus oreadorum* | Arctic | Glacial ice | Pink to reddish | Intracellular | Carotenoids | Not described | Turchetti et al. (2018) |
| *Sporobolomyces roseus* | Antarctica (Livingston Island) | Soil | Reddish-pink | Intracellular | Possibly carotenoids | Not described | Davoli & Webe (2002); Rusinova-Videva et al. (2024) |
